# Proteomic Dynamics Reveal Cell-Cycle and Rho GTPase Remodeling Associated with Transient Senescence Traits in Human Chondrocytes During Sustained IL-1β Signaling

**DOI:** 10.3390/cells15161431

**Published:** 2026-08-08

**Authors:** Hellen Paula Valerio, Thatiana Corrêa de Melo, Mariana Barbosa de Souza Rizzo, Amanda Teixeira de Melo, Miryam Paola Alvarez-Flores, Ana Marisa Chudzinski-Tavassi

**Affiliations:** Centre of Excellence in New Target Discovery (CENTD), Butantan Institute, São Paulo 05503-900, Brazil; thatiana.melo@butantan.gov.br (T.C.d.M.); m.rizzo.proppg@proppg.butantan.gov.br (M.B.d.S.R.); a.melo.proppg@proppg.butantan.gov.br (A.T.d.M.); miryam.flores@butantan.gov.br (M.P.A.-F.)

**Keywords:** chondrocytes, IL-1β, Interleukin-1β, proteomics, cell cycle, Rho GTPases, senescence, chronic inflammation

## Abstract

**Highlights:**

**What are the main findings?**

- Time-resolved proteomics revealed progressive, exposure-duration-associated remodeling of protein networks in an in vitro model of primary human articular chondrocytes exposed to sustained IL-1β stimulation.
- Sustained IL-1β exposure was associated with growth arrest and transient senescence-associated traits, together with coordinated abundance changes in cell-cycle regulators and Rho GTPase-associated cytoskeletal proteins.

**What are the implications of the main findings?**

- The identified cell-cycle and Rho GTPase-associated proteomic signatures provide candidate mechanisms for future investigation of how sustained inflammatory signaling affects chondrocyte function.
- BrdU measurement showed partial recovery of DNA synthesis following cytokine withdrawal, suggesting some degree of proliferative plasticity without establishing reversibility of the broader cellular phenotype.

**Abstract:**

Chronic inflammatory signaling contributes to cartilage degeneration across multiple joint diseases, yet the molecular consequences of sustained cytokine exposure remain incompletely understood. We investigated how prolonged interleukin-1β (IL-1β) stimulation remodels the chondrocyte proteome and whether these changes are associated with senescence-associated traits. Primary human articular chondrocytes were exposed to IL-1β (10 ng/mL) for up to four days. Time-resolved data-independent acquisition (DIA) proteomics was integrated with immunofluorescence, quantitative PCR, multiplex metalloproteinase profiling, BrdU incorporation, growth-curve analysis, and senescence-associated β-galactosidase assays. Sustained IL-1β induced extensive time-dependent proteomic remodeling, with early inflammatory and extracellular matrix responses followed by alterations in cell-cycle regulation and cytoskeletal organization. Prolonged stimulation was associated with persistent downregulation of CDK4, Cyclin D1, DNA replication-associated proteins, and Rho GTPase-associated components, accompanied by actin cytoskeletal remodeling. These molecular changes were associated with impaired proliferation, increased senescence-associated β-galactosidase activity, and transient modulation of p21. Following cytokine withdrawal, BrdU incorporation showed partial recovery. Together, these findings indicate that sustained IL-1β progressively reshapes the chondrocyte cellular state through coordinated remodeling of proliferative, cytoskeletal, and metalloprotease programs while showing some degree of proliferative plasticity under the conditions tested.

## 1. Introduction

Chronic inflammation is a central driver of tissue damage and disease progression in several joint disorders, including rheumatoid arthritis and osteoarthritis [1,2]. Among the cytokines implicated in these conditions, interleukin-1β (IL-1β) is a key mediator of tissue remodeling and inflammatory signaling [3]. IL-1β is produced by synovial cells, infiltrating immune cells, and chondrocytes, and exerts pleiotropic effects on cartilage [4]. Sustained IL-1β activity is thought to contribute to the dysregulation of chondrocyte and cartilage homeostasis and has therefore been widely employed as an experimental model of inflammatory stress in chondrocytes [5,6]. Consistent with its pathogenic role, IL-1β has also emerged as a therapeutic target in several inflammatory and autoinflammatory diseases [7].

Exposure of chondrocytes to IL-1β induces catabolic responses characterized by increased expression and secretion of several matrix metalloproteinases and other factors involved in cartilage breakdown [8]. Beyond its well-established role in cartilage catabolism, IL-1β has been shown to influence fundamental aspects of chondrocyte biology [9]. Transcriptomic studies have demonstrated that IL-1β induces broad changes in gene expression, affecting processes related to cell survival, proliferation, and cytoskeleton organization [10,11,12,13,14].

Recent studies have proposed that sustained inflammatory signaling induced by IL-1β could promote senescence-associated traits in chondrocytes, including impaired proliferation, increased expression of cell-cycle regulators such as p21 and p16^INK4a^, enhanced senescence-associated β-galactosidase activity, and alterations in secretory activity [15,16,17,18]. However, many of these studies evaluated relatively limited panels of senescence-associated markers. As no single marker is sufficient to define cellular senescence, distinguishing an established senescent phenotype from other forms of stress-associated or reversible growth arrest requires the combined assessment of multiple molecular, morphological, and functional features [19]. Consequently, evidence remains conflicting. While some reports describe the emergence of senescence-associated phenotypes following chronic IL-1β exposure, others failed to detect canonical hallmarks of irreversible senescence, suggesting instead a stress-adaptive state that only partially overlaps with established senescence programs [20]. These discrepancies raise an important question regarding the nature of the cellular state that emerges during prolonged inflammatory stimulation. Distinguishing a reversible inflammatory adaptation from a more persistent senescence-associated state may be clinically relevant, as these phenotypes may differ in their capacity for recovery and responsiveness to therapeutic intervention.

Proteomic approaches have provided important insights into the molecular effects of IL-1β in chondrocytes, revealing alterations in mitochondrial proteins, extracellular matrix turnover, endoplasmic reticulum stress responses, and secreted inflammatory mediators [21,22,23]. However, these studies have predominantly focused on acute stimulation periods of 24–48 h and relied on proteomic approaches such as two-dimensional gel electrophoresis and data-dependent acquisition (DDA) mass spectrometry, which provide more limited proteome coverage and quantitative reproducibility than current data-independent acquisition (DIA) workflows [24].

As a result, little is known about how sustained inflammatory signaling reshapes the global chondrocyte proteome over time and whether progressive proteomic remodeling converges on senescence-associated programs. To address this gap, we applied time-resolved DIA proteomics, which enables deep and consistent proteome profiling across multiple time points, combined with complementary molecular and cellular assays to characterize the temporal trajectory of proteomic remodeling induced by sustained IL-1β exposure in primary human chondrocytes. By integrating global protein expression changes with complementary cellular phenotyping, we sought to define the molecular landscape associated with prolonged inflammatory stress and determine whether it is accompanied by the emergence of senescence-associated signatures. The study was therefore designed as a discovery-oriented investigation aimed at identifying exposure-duration-associated molecular signatures and candidate pathways for future mechanistic and translational studies.

## 2. Materials and Methods

### 2.1. Experimental Model

This study was designed as an in vitro time-course investigation in which primary human articular chondrocytes derived from 2 non-diseased donors were exposed to IL-1β (10 ng/mL) for 24, 48, 72, or 96 h, followed by time-resolved DIA proteomics and complementary molecular and cellular assays.

Commercial primary Normal Human Articular Knee Chondrocytes (NHAC-Kn) were obtained from Lonza (#CC-2550, Walkersville, MD, USA) and cultured in DMEM/F-12 Ham’s medium (#11320033; Gibco, Bleiswijk, The Netherlands) supplemented with 15 mM HEPES (#1003098482; Sigma-Aldrich, Saint Louis, MO, USA), 10% fetal bovine serum (FBS; #12657029; Gibco, São Paulo, Brazil), 100 U/mL penicillin, and 100 µg/mL streptomycin (#15140122; PenStrep, Gibco, Grand Island, NY, USA). Cells were maintained at 37 °C in a humidified atmosphere with 5% CO_2_. Cells were routinely tested for mycoplasma contamination.

For IL-1β treatment, cells were exposed to 10 ng/mL of IL-1β (R&D Systems #201-LB-025, Minneapolis, MN, USA) diluted in DMEM/F12 containing 10% FBS, with daily medium replacement and cytokine replenishment. Control cells were cultured in the identical serum-supplemented medium without IL-1β for the same duration. For all experiments, quiescent, contact-inhibited control cells maintained for 96 h were used as the reference condition to account for differences in cell density, proliferative status, and potential time-in-culture effects independent of IL-1β treatment, thereby facilitating the interpretation of treatment-induced molecular changes associated with growth arrest and senescence-related remodeling.

Importantly, all experimental groups were seeded on the same day and harvested simultaneously after a total of 96 h in culture. IL-1β exposure was initiated in a staggered manner so that cells were treated for 96, 72, 48, or 24 h before the common collection time. Specifically, the 96 h treatment was initiated on the first experimental day, followed by the 72 h, 48 h, and 24 h treatments on each subsequent day. Untreated control cells were maintained for the complete 96 h period under identical culture and daily medium-replacement conditions without IL-1β. Therefore, all groups had the same total time in culture and differed in the duration of cytokine exposure.

For the BrdU incorporation assay, an additional untreated early-passage control analyzed after 24 h (CTL− 24 h) was included to represent actively proliferating chondrocytes, whereas untreated cells maintained under contact-inhibited conditions for 96 h (CTL− 96 h) served as the quiescent reference condition. Cells from two donors were used for the proteomic analysis. Complementary assays were performed using cells from one of these donor lots, as specified in the corresponding figure legends; therefore, they provide orthogonal corroboration within this experimental model rather than independent validation across donors. Unless otherwise specified, the n values reported in the figure legends refer to independent culture-level experiments in which cells were separately cultured, treated, harvested, and processed. For the proteomic analysis, six independent culture-level experiments were performed per condition, comprising three independently established cultures from each donor. No technical replicates, such as repeated sample preparation or repeated LC–MS/MS analysis of the same cell lysate, were included. The only exception was the qRT-PCR assay, for which three independent culture-level experiments were analyzed in technical duplicate. All experiments were performed using cells at passage 3 (P3) to ensure consistency and to minimize the contribution of replicative senescence. As reported in the supplementary data of a previous work by our group [25], where the same lot of cells from the same donors have been employed, the COL2A1/COL1A1 ratio serves as a key indicator of the preserved chondrogenic phenotype in these cells at passage 3, even though some level of dedifferentiation is known to affect chondrocytes passaged in monolayer culture, imposing a limitation in this type of in vitro model.

### 2.2. Sample Preparation for Proteomic Analysis

For proteomics experiments, treated and control cells were cultured in 6-well plates. Cells were then washed three times with phosphate-buffered saline (PBS) and scraped into 200 µL of lysis buffer containing 100 mM ammonium bicarbonate (#09830, Lot #BCBL6295V, Sigma-Aldrich, Steinheim, Germany) and 1% sodium deoxycholate (#30970, Lot # BCBN9392V, St. Louis, MO, USA). Cell lysis was performed on ice to minimize protein degradation by intracellular proteases. Protein concentration was determined using a Qubit fluorometer (#Q33216, Thermo Fisher Scientific, Eugene, OR, USA) on diluted samples, according to the manufacturer’s instructions.

### 2.3. Protein Digestion

For cell lysates, 60 µg of protein per sample was subjected to reduction, alkylation, and enzymatic digestion. Reduction was performed using 10 mM dithiothreitol (DTT) (#V3151, Promega, Madison, WI, USA) for 1 h at 30 °C with agitation at 800 rpm. Proteins were subsequently alkylated with 15 mM iodoacetamide (#I1149-5G, Sigma Aldrich, St. Louis, MO, USA) in the dark for 30 min at room temperature. Proteolytic digestion was carried out overnight at 37 °C with agitation (800 rpm) by addition of trypsin (#V5111, Lot #0000667238, Promega, Madison, WI, USA) at a 1:40 (*w*/*w*) trypsin-to-protein ratio. Digestion was stopped by adding trifluoroacetic acid (TFA; #T6508, Lot #STBJ4083, Sigma-Aldrich, St. Louis, MO, USA) to a final concentration of 1%. Peptides were subsequently desalted using in-house-prepared StageTips packed with C18 membranes (Supelco, #66883-U, Lot #81840, Bellefonte, PA, USA) and vacuum-dried using a centrifugal concentrator (Eppendorf, Enfield, CT, USA). Briefly, samples were washed ten times with 1% TFA and eluted with 50% acetonitrile (#741857, Sigma-Aldrich, St. Louis, MO, USA). Finally, peptides were reconstituted in 20 µL of aqueous buffer containing 0.1% formic acid (#94318, Lot #BCBP4740V, Sigma-Aldrich, Steinheim, Germany) for LC–MS/MS analysis.

### 2.4. Liquid Chromatography–Tandem Mass Spectrometry (LC-MS/MS) Analysis

Peptide samples were analyzed using an Orbitrap Exploris 240 mass spectrometer (Thermo Fisher Scientific, Bremen, Germany) coupled to a Vanquish Neo nano-liquid chromatography (nano-LC) system (Thermo Fisher Scientific) equipped with an Easy-Spray™ ion source. Peptides were separated on a reversed-phase Acclaim™ PepMap™ analytical column (150 mm × 75 μm inner diameter, 2 μm particle size) in combination with an Acclaim™ PepMap™ 100 C18 trap column (20 mm × 75 μm, 3 μm), both from Thermo Fisher Scientific. Peptides were eluted using a linear gradient of 2–40% solvent B (80% acetonitrile, 0.1% formic acid) over 130 min at a flow rate of 300 nL/min, with the electrospray voltage set to 1.5 kV. Mass spectra were acquired using a DIA strategy. Full MS (MS1) scans were acquired at a resolution of 60,000 over a mass range of *m*/*z* 400–900 with an automatic gain control (AGC) target of 300% and automatic maximum injection time. DIA MS/MS scans were acquired at a resolution of 15,000 using sequential 10 *m*/*z* isolation windows covering the full precursor mass range, with an AGC target of 400%, a maximum injection time of 27 ms, and a normalized collision energy of 30%.

### 2.5. Proteomic Data Processing and Statistical Analysis

DIA data were processed and analyzed using DIA-NN (v2.2.0) for peptide identification and quantification. Peptide sequence searches were performed against the human SwissProt database (20,399 entries; accessed December 2025). In silico spectral libraries were generated from the reference database, incorporating predicted retention times. A maximum of two missed cleavages was allowed, and peptide lengths were restricted to 7–30 amino acids. Up to two variable modifications were permitted, including methionine oxidation and methionine loss at the protein N-terminus, while carbamidomethylation of cysteine was specified as a fixed modification. To reduce missing values across samples, the match between runs option was enabled, and quantification was performed using the QuantUMS (high-precision) strategy. Mass tolerance was automatically optimized by DIA-NN. Peptide and protein identifications were filtered at a false discovery rate (FDR) of 1%. Downstream proteomics data analysis was performed in R (v4.1.3). Differential protein abundance was assessed using the limma Bioconductor package (v3.50.3) with an empirical Bayes-moderated *t*-test and donor identity included as a blocking factor. Resulting *p*-values were adjusted for multiple testing using the Benjamini–Hochberg (BH) method. Gene Ontology enrichment analysis was conducted using clusterProfiler (v4.2.2). Proteins were considered differentially regulated when they exhibited a Benjamini–Hochberg-adjusted *p*-value below 0.05 and an absolute log_2_ fold change greater than 0.5.

Proteins exhibiting significant abundance differences across the experimental conditions were identified by one-way analysis of variance, followed by false discovery rate correction using the Benjamini–Hochberg method. Proteins with an adjusted *p*-value below 0.05 were retained for temporal clustering. For each protein, LFQ intensity values were log_2_-transformed, and the median abundance was calculated for each experimental condition. The resulting protein-by-condition matrix was standardized by calculating row-wise z-scores. Proteins with incomplete or non-finite standardized profiles were excluded. Hierarchical clustering was performed using Euclidean distance and complete-linkage agglomeration. The resulting dendrogram was divided into six clusters using the cutree function in R. The number of clusters was set to six to summarize the major temporal expression patterns. GO Biological Process enrichment was used to capture broad functional changes at individual time points. In contrast, Reactome pathway analysis was applied to temporally clustered proteins to enable mechanistic interpretation of dynamic pathway regulation.

### 2.6. Senescence-Associated β-Galactosidase (SA-β-Gal) Staining

To assess SA-β-gal activity, NHAC-Kn chondrocytes were continuously exposed to IL-1β (10 ng/mL) in serum-supplemented medium for 96 h, with daily medium replacement and cytokine replenishment. Cells were then fixed in 4% paraformaldehyde (PFA), washed three times with PBS, and stained using a Senescence β-Galactosidase Staining Kit (Cell Signaling Technology, #9860, Danvers, MA, USA) according to the manufacturer’s instructions. SA-β-gal-positive cells were visualized and imaged by phase-contrast microscopy.

SA-β-gal staining was quantified using ImageJ/Fiji software (version 1.54r). For each experimental condition, three randomly selected, non-overlapping fields were analyzed per independent experiment. Individual cells were segmented based on visible cell boundaries, and blue cytoplasmic staining was detected using a predefined color threshold applied uniformly to all images. Cells exhibiting blue cytoplasmic staining above the background observed in untreated controls were classified as SA-β-gal positive. The percentage of SA-β-gal-positive cells was calculated relative to the total number of segmented cells in each field. At least 300 cells were analyzed per condition across three independent experiments. Image analysis was performed with the investigator blinded to the experimental conditions. Statistical significance was determined using Student’s T test.

### 2.7. Growth Curve Assay

A growth curve assay was performed to assess the long-term effects of sustained IL-1β exposure on chondrocyte proliferation. NHAC-Kn cells were seeded in 25 cm^2^ culture flasks at an initial density of 0.5 × 10^5^ cells per flask and continuously exposed to IL-1β (10 ng/mL) throughout the 15-day experimental period. Culture medium and IL-1β were replenished as described above. At the indicated time points, cells were harvested and counted using a Neubauer hemocytometer. Cell viability was determined by trypan blue exclusion. Cell morphology and confluence were monitored throughout the experiment using an inverted microscope at 10× magnification.

### 2.8. Immunofluorescence Staining and High-Content Screening Analysis

NHAC-kn cells were seeded in black 96-well plates compatible with High-Content Screening (HCS) from Greiner Bio-One, PP Microplate, 96 well, catalog no. 650261, sterile, at a density of 0.8 × 10^4^ cells per well. After cell adhesion, P3 cultures were chronically treated with IL-1β at 10 ng/mL and evaluated at 24 h, 48 h, 72 h, and 96 h. Untreated P3 cells were maintained in culture for 96 h as the negative control (CTL−). After each treatment time point, cells were washed with PBS and fixed with 4% PFA for 20 min at room temperature. The cells were then washed again with PBS and permeabilized with 0.1% Triton X-100 for 15 min at 4 °C. After permeabilization, nonspecific binding sites were blocked with PBS containing 3% Bovine Serum Albumin (BSA) for 60 min. For immunofluorescence staining, cells were incubated overnight with the primary antibodies diluted in blocking solution. The senescence-associated marker p21 was detected using a recombinant rabbit monoclonal anti-p21 antibody (clone 2H2L13, catalog no. 701151, Invitrogen/Thermo Fisher Scientific, Waltham, MA, USA) at a dilution of 1:400. RhoA protein was detected using a mouse monoclonal anti-RhoA antibody (clone 26C4, catalog no. sc-418, Santa Cruz Biotechnology, Dallas, TX, USA) at a dilution of 1:100. After primary antibody incubation, cells were washed three times with PBS and incubated with the corresponding Alexa Fluor 647-conjugated anti-mouse secondary antibodies (Invitrogen/Thermo Fisher Scientific, Waltham, MA, USA) at a dilution of 1:1000, protected from light. Secondary-only controls for anti-rabbit Alexa Fluor 647 and anti-mouse Alexa Fluor 647 were included to evaluate nonspecific background staining. In parallel, the organization of the actin cytoskeleton was assessed using Alexa Fluor 488 Phalloidin (catalog no. A12379, Invitrogen/Thermo Fisher Scientific, Waltham, MA, USA) at a dilution of 1:400. Nuclei were counterstained with Hoechst 33342 (catalog no. H1399, Molecular Probes/Invitrogen/Thermo Fisher Scientific, Eugene, OR, USA) at a final concentration of 0.1 µg/mL. After staining, cells were maintained in PBS and protected from light until image acquisition. Images were acquired using the ImageXpress^®^ Micro Confocal High-Content Imaging System (Molecular Devices, San Jose, CA, USA), applying identical exposure time, camera gain, illumination intensity, and acquisition settings for all experimental conditions within each fluorescence channel. Representative immunofluorescence images were obtained for F-actin, stained with Alexa Fluor™ 488-conjugated Phalloidin (green), and nuclei stained with Hoechst 33342 (blue). For High-Content Screening (HCS) analyses, sixteen non-overlapping fields per well were automatically acquired using a 20× objective according to a predefined acquisition map. Approximately 2500–3000 cells were automatically segmented and analyzed per well across the sixteen acquired fields, depending on cell density. Image segmentation and quantitative analyses were performed using MetaXpress^®^ Acquisition & Analysis Software version 6.7.2.290 (Molecular Devices, San Jose, CA, USA), integrated with the ImageXpress^®^ Micro Confocal High-Content Imaging System (Molecular Devices, San Jose, CA, USA). Nuclear segmentation was based on Hoechst 33342 fluorescence, which served as the primary object for cell identification, followed by automated cytoplasmic mask generation according to the respective fluorescence channel. Quantitative measurements were automatically extracted from all segmented cells within each field. Positivity thresholds and fluorescence intensity cutoffs were established using untreated negative controls and background fluorescence measured in unstained control wells. The same acquisition settings, segmentation parameters, fluorescence thresholds, and classification criteria were applied to all experimental groups. Image acquisition and quantitative analyses were conducted using predefined acquisition and analysis protocols, without manual field selection or post-acquisition modification of the analysis parameters. The resulting quantitative data were exported for subsequent statistical analysis.

### 2.9. BrdU Incorporation Assay and Analysis of Cell Proliferation by High-Content Screening

Cell proliferation was evaluated by 5-bromo-2′-deoxyuridine (BrdU) (catalog number B5002, Sigma Aldrich/Merck, St. Louis, MO, USA) incorporation assay in NHAC-kn cells subjected to sustained exposure to IL-1β. Passage 3 cells (P3) were maintained in culture for 96 h and used as the negative control. NHAC-kn cells were seeded in black 96-well plates compatible with HCS from Greiner Bio-One, PP microplate, catalog no. 650261 (Frickenhausen, Baden-Württemberg, Germany), at a density of 0.8 × 10^4^ cells per well, following the same experimental conditions used for the immunofluorescence assays. After cell adhesion, P3 cultures were chronically treated with IL-1β at a concentration of 10 ng/mL of IL-1β and evaluated at 24 h, 48 h, 72 h, and 96 h. For the cytokine-withdrawal condition, cells were exposed to IL-1β for 96 h, washed, and maintained for an additional 48 h in cytokine-free complete medium before the final 4 h BrdU pulse. For each experimental time point, the BrdU pulse was performed on the corresponding collection day. BrdU was added directly to the culture medium at a final concentration of 50 µM and incubated for 4 h at 37 °C in a humidified atmosphere containing 5% CO_2_. After the incorporation period, the BrdU-containing medium was removed, and the cells were washed twice with PBS and immediately fixed with 4% PFA for 20 min at room temperature. For the plates corresponding to the 24 h, 48 h, 72 h, and 96 h time points, BrdU was added 4 h before fixation. For the 96 h treatment condition, the culture medium was removed and replaced with fresh medium, and the cells were maintained for 48 h before the final 4 h BrdU pulse. After this period, the cells were washed twice with PBS and fixed with 4% PFA. After fixation, the cells were washed with PBS and permeabilized with 0.1% Triton X-100 for 15 min at 4 °C. To allow exposure of the BrdU incorporated into DNA, the cells were incubated with 2 M HCl for 10 min at room temperature. After acid denaturation, the HCl solution was removed, and the cells were neutralized with 0.1 M sodium borate buffer, pH 8.5, for 5 min. The cells were then washed with PBS to completely remove the neutralization buffer. After the denaturation/neutralization step, nonspecific binding sites were blocked with PBS containing 3% BSA for 60 min. Detection of incorporated BrdU was performed by overnight incubation with an anti-BrdU antibody conjugated to PerCP-CyTM 5.5 (BD PharmingenTM, clone 3D4, catalog number 560809, San Jose, CA, USA), at a dilution of 1:800 in blocking solution. The plate was kept protected from light throughout the incubation step with the conjugated antibody. After overnight incubation, the cells were washed three times with PBS to remove unbound antibodies. Nuclei were counterstained with Hoechst 33342 at 5 µg/mL to allow nuclear segmentation and quantification of the total number of cells by HCS. The cells were maintained in PBS and protected from light until image acquisition.

Images were acquired using a High-Content Screening platform, applying standardized exposure, gain, and intensity parameters for all experimental conditions. BrdU fluorescence labeled with PerCP-Cy5.5 was acquired in the Cy5-compatible channel. Quantitative analysis was performed by nuclear segmentation based on Hoechst staining and quantification of the percentage of BrdU-positive cells relative to the total number of nuclei per field/well. The results were expressed as the percentage of BrdU-positive cells.

### 2.10. Real-Time Quantitative Polymerase Chain Reaction (qRT-PCR)

Total RNA was extracted from control and treated cells using TRIzol™ Reagent (Invitrogen, Carlsbad, CA, USA, Cat. No. 15596026). Reverse transcription was performed using 500 ng of total RNA with the SuperScript™ IV First-Strand Synthesis System (Invitrogen, Cat. No. 18091050) according to the manufacturer’s instructions. Quantitative real-time PCR (qRT-PCR) was carried out in a final reaction volume of 8 µL, containing 4 µL of Fast SYBR™ Green Master Mix (Applied Biosystems™, Vilnius, Lithuania, Cat. No. #4385612), 1 µL of forward primer (400 nM), 1 µL of reverse primer (400 nM), and 2 µL of 1:10 diluted cDNA (5 ng). Primer sequences are detailed in Table S1. All qRT-PCR reactions were performed in technical duplicate using the QuantStudio™ 3 Real-Time PCR System (Applied Biosystems™). Relative mRNA expression was calculated using the comparative ΔΔCt method. Briefly, the Ct obtained for each target gene was normalized to the mean of two endogenous control genes (B2M and RPL37A) for each culture-level replicate (ΔCt = Ct target − Ct mean of controls). The ΔΔCt was subsequently calculated by subtracting the average ΔCt of the reference control group from the ΔCt of each sample (ΔΔCt = ΔCt treated sample − ΔCt mean of reference sample). Relative quantity (RQ) values were calculated using the formula 2^−ΔΔCt^.

### 2.11. Cytokine and Metalloprotease Release Analysis

Chondrocytes were seeded in 96-well plates and exposed to IL-1β (10 ng/mL) as described for the other experiments. At the indicated time points, cell-free conditioned media were collected and stored at −80 °C until analysis. Cytokine concentrations were quantified using the MILLIPLEX^®^ MAP Human Cytokine/Chemokine Magnetic Bead Panel (Millipore, #HCYTOMAG-60K-05, Burlington, MA, USA), according to the manufacturer’s instructions. The panel included IL-6, IL-8, IL-10, and TNF-α. Matrix metalloproteinases (MMP-1, MMP-2, MMP-7, MMP-9, MMP-10) were measured using the MILLIPLEX^®^ MAP Human MMP Magnetic Bead Panel 2 (Millipore, #HMMP2MAG-55K, Burlington, MA, USA) and MMP13 using the Panel 1 (Millipore, #HMMP1MAG-55K, Burlington, MA, USA). Data acquisition was performed using xPONENT^®^ 4.3 software (Luminex Corporation, Austin, TX, USA), and analyte concentrations were calculated with MILLIPLEX^®^ Analyst 5.1 software.

Cytokine and MMP concentrations were measured in conditioned medium and reported as pg/mL without normalization to cell number. Cell numbers were assessed in parallel by automated high-content imaging based on the quantification of Hoechst-positive nuclei. No statistically significant differences in nuclear counts were observed among the experimental conditions. Because the medium was replaced daily, the concentrations measured at each endpoint reflected factor release during the interval since the most recent medium replacement rather than cumulative secretion over the entire treatment period.

## 3. Results

### 3.1. Time-Resolved Proteomics Reveals Coordinated and Temporally Distinct Responses to Sustained IL-1β Exposure

**Figure 1:**
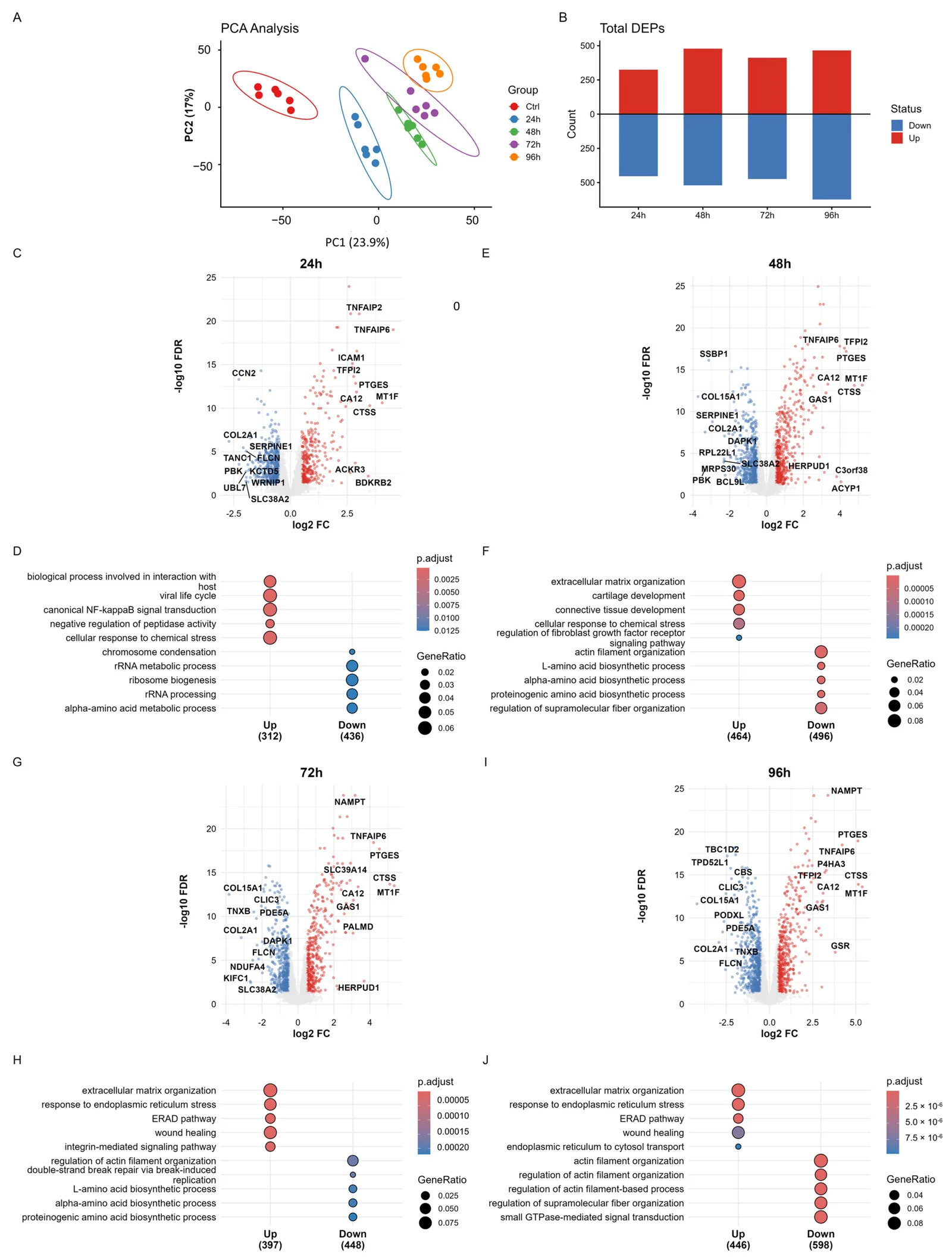
Prolonged IL-1β stimulation induces widespread proteomic remodeling in human chondrocytes. (**A**) Principal component analysis (PCA) showing the separation of control and treated samples over time (24 h, 48 h, 72 h, and 96 h) (*n* = 6 independent culture-level experiments per condition, comprising three independently established cultures from each of two donors). (**B**) Total number of differentially expressed proteins (DEPs) at each time point, distinguishing upregulated and downregulated proteins. (**C**,**E**,**G**,**I**) Volcano plots illustrating differentially expressed proteins at 24 h, 48 h, 72 h, and 96 h, respectively. Quiescent, untreated chondrocytes under contact-inhibited conditions, cultured for 96 h, were used as controls in this experiment. Differentially expressed proteins were defined using FDR < 0.05 and |log2FC| > 0.5 using a limma model. (**D**,**F**,**H**,**J**) Gene Ontology (GO) enrichment analysis of upregulated and downregulated proteins at each time point, highlighting biological processes associated with cellular stress responses, metabolism, and extracellular matrix organization.

First, we assessed whether exposure to 10 ng/mL of IL-1β, a concentration commonly used in the literature [15,16,17,18,26] and selected to reproduce robust inflammatory stimulation, induces sustained proteomic changes in primary chondrocytes over four days. Quiescent, contact-inhibited control cells cultured for 96 h were used as the reference condition to account for differences in cell density, proliferative status, and potential time-in-culture effects independent of IL-1β treatment, facilitating the interpretation of proteomic changes potentially associated with growth arrest and senescence-associated remodeling. Principal component analysis revealed a treatment-dependent effect and time-dependent clustering of samples, indicating that chronic IL-1β exposure dynamically reshapes the chondrocyte proteome over time (Figure 1A). Notably, the 24 h samples formed a distinct cluster separated from later time points, whereas the 48 h and 72 h samples occupied an intermediate position, suggesting progressive, exposure-duration-associated remodeling of the chondrocyte proteome during sustained IL-1β stimulation. Across the 5500 proteins quantified, between 779 and 1089 proteins met the differential-abundance criteria of FDR < 0.05 and |log2FC| > 0.5 at each time point (Figure 1B). Differential expression analysis revealed a predominance of down-regulated proteins throughout the time course, particularly at later time points. Detailed quantification per protein and condition, along with the statistical results of the limma analysis, are available in Supplementary Spreadsheet S1.

The proteins most strongly regulated at each time point are shown in the volcano plots (Figure 1C,E,G,I). Among the proteins consistently up-regulated throughout the experiment were TNFAIP2, TNFAIP6, PTGES, and CTSS, all of which have previously been reported to be induced by IL-1β [27,28,29,30]. TNFAIP2 and TNFAIP6 are inducible inflammatory mediators commonly associated with IL-1β-driven responses [27,28], whereas PTGES catalyzes the terminal step of prostaglandin E2 synthesis, a major effector pathway regulated by IL-1β in chondrocytes [29,31]. CTSS, a lysosomal cysteine protease implicated in extracellular matrix degradation, was also strongly induced [32]. In contrast, several proteins involved in cartilage homeostasis and extracellular matrix maintenance were consistently down-regulated, including COL2A1, the principal collagen component of articular cartilage, as well as CCN2, a matricellular protein involved in cartilage homeostasis and matrix synthesis, and COL15A1, a basement membrane-associated collagen implicated in extracellular matrix organization [33,34,35]. Together, these changes highlight a sustained shift from a cartilage-maintaining to an inflammatory and tissue-remodeling proteomic program.

To gain insight into the biological processes underlying these proteomic changes, we performed functional enrichment analysis on differentially expressed proteins at each time point (Figure 1D,F,H,J). Early responses were dominated by pathways associated with inflammatory signaling, cellular responses to chemical stress, oxidative stress, and NF-κB activation. As stimulation progressed, enrichment patterns shifted toward processes involved in extracellular matrix organization, endoplasmic reticulum stress, DNA replication, cytoskeletal regulation, and small GTPase signaling. These findings indicate that prolonged IL-1β exposure extends beyond the initial inflammatory response and progressively impacts pathways involved in cellular organization, protein homeostasis, and proliferative capacity. Supplementary Spreadsheet S2 containing the genes associated with each enriched process is available in the Supplementary Files section.

**Figure 2:**
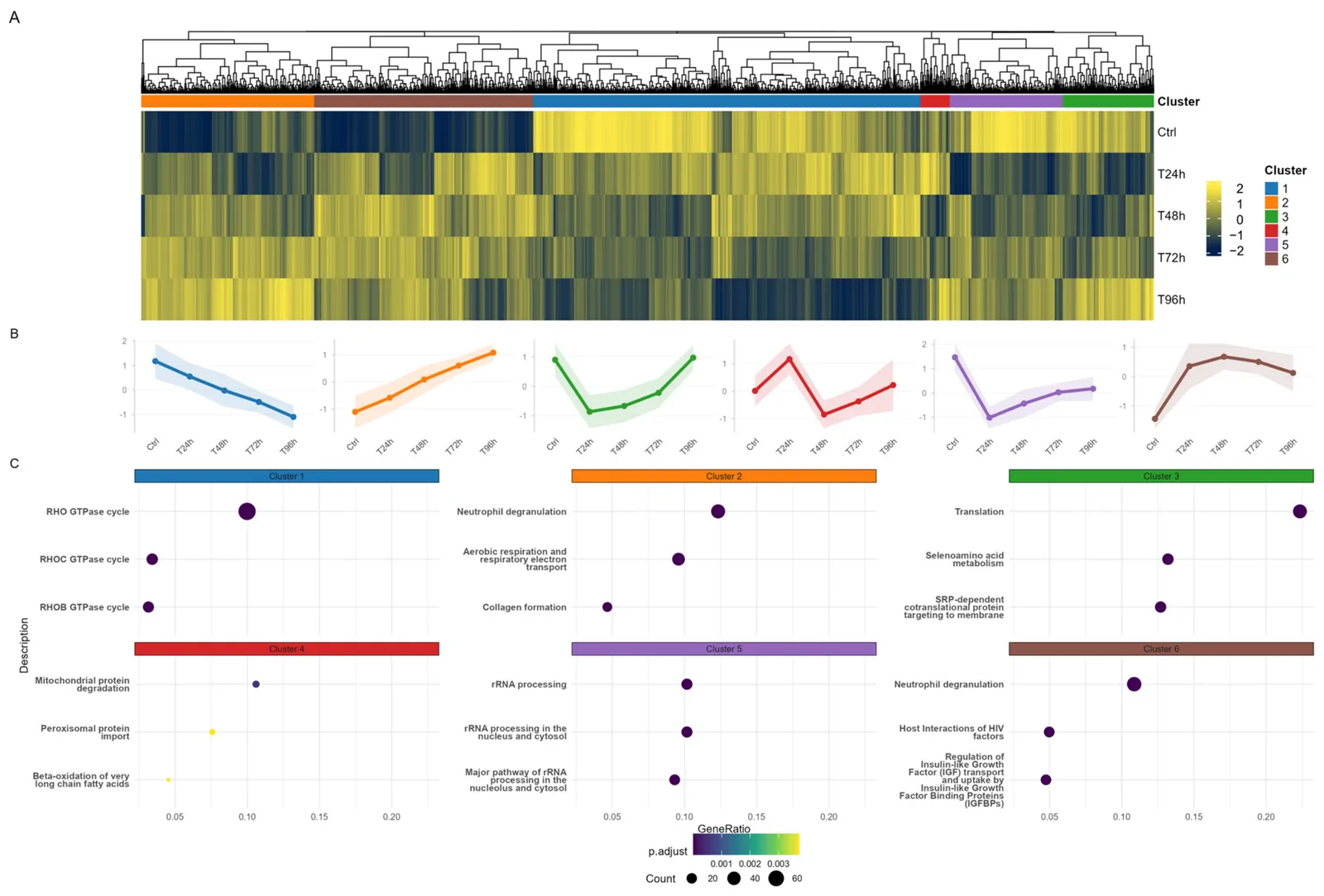
Temporal clustering reveals coordinated proteomic programs induced by prolonged IL-1β stimulation. (**A**) Hierarchical clustering and heatmap of differentially expressed proteins across control, 24 h, 48 h, 72 h, and 96 h conditions (*n* = 6 independent culture-level experiments per condition, comprising three independently established cultures from each of two donors). Quiescent, untreated chondrocytes under contact-inhibited conditions, cultured for 96 h, were used as controls in this experiment. Each column represents the median intensity of proteins for a given biological condition. Proteins are grouped into six clusters based on their temporal expression patterns. (**B**) Median expression profiles are shown for each cluster to illustrate dynamic trends over time. Shaded regions denote the interquartile range, reflecting the variability within each cluster. (**C**) Functional enrichment analysis of each cluster, based on Reactome’s terms. Dot size represents the number of proteins per pathway, and color indicates adjusted *p*-values. The x axis represents the gene ratio.

To identify specific coordinated proteomic programs induced by prolonged IL-1β stimulation in chondrocytes, proteins significantly regulated across all experimental groups (FDR-corrected ANOVA) were subjected to hierarchical clustering. This analysis revealed six major temporal expression profiles (Figure 2A; detailed ANOVA results are provided in Supplementary Spreadsheet S3). Each cluster displayed a distinct pattern of regulation over time, reflecting the dynamic nature of the chondrocyte response to prolonged inflammatory stimulation (Figure 2B). Among these, two clusters exhibited progressive behavior, with proteins showing either continuous down-regulation (Cluster 1, shown in Figure 2A,B) or up-regulation (Cluster 2, Figure 2A,B) across the four-day stimulation period, while the remaining clusters displayed transient, delayed, or biphasic responses.

Reactome pathway enrichment analysis revealed functionally coherent modules associated with each temporal pattern (Figure 2C). The progressively down-regulated cluster was enriched for Rho GTPase-mediated signaling pathways, including RHOC, RHOB, and RHO GTPase cycles, suggesting sustained perturbation of cytoskeletal regulatory networks. In contrast, the progressively up-regulated cluster was enriched for extracellular matrix organization, inflammatory pathways, and aerobic respiration. Additional clusters were associated with translation-related processes, mitochondrial and peroxisomal metabolism, rRNA processing, and growth factor-regulated pathways. The complete protein lists associated with each cluster are provided in Supplementary Spreadsheet S4. Collectively, these analyses indicate that sustained IL-1β exposure triggers an early inflammatory and stress-response program that progressively evolves into broader remodeling of cytoskeletal, metabolic, and homeostatic pathways.

### 3.2. Cell-Cycle and Cytoskeletal Remodeling Are Associated with the Emergence of Senescence-Associated Traits

**Figure 3:**
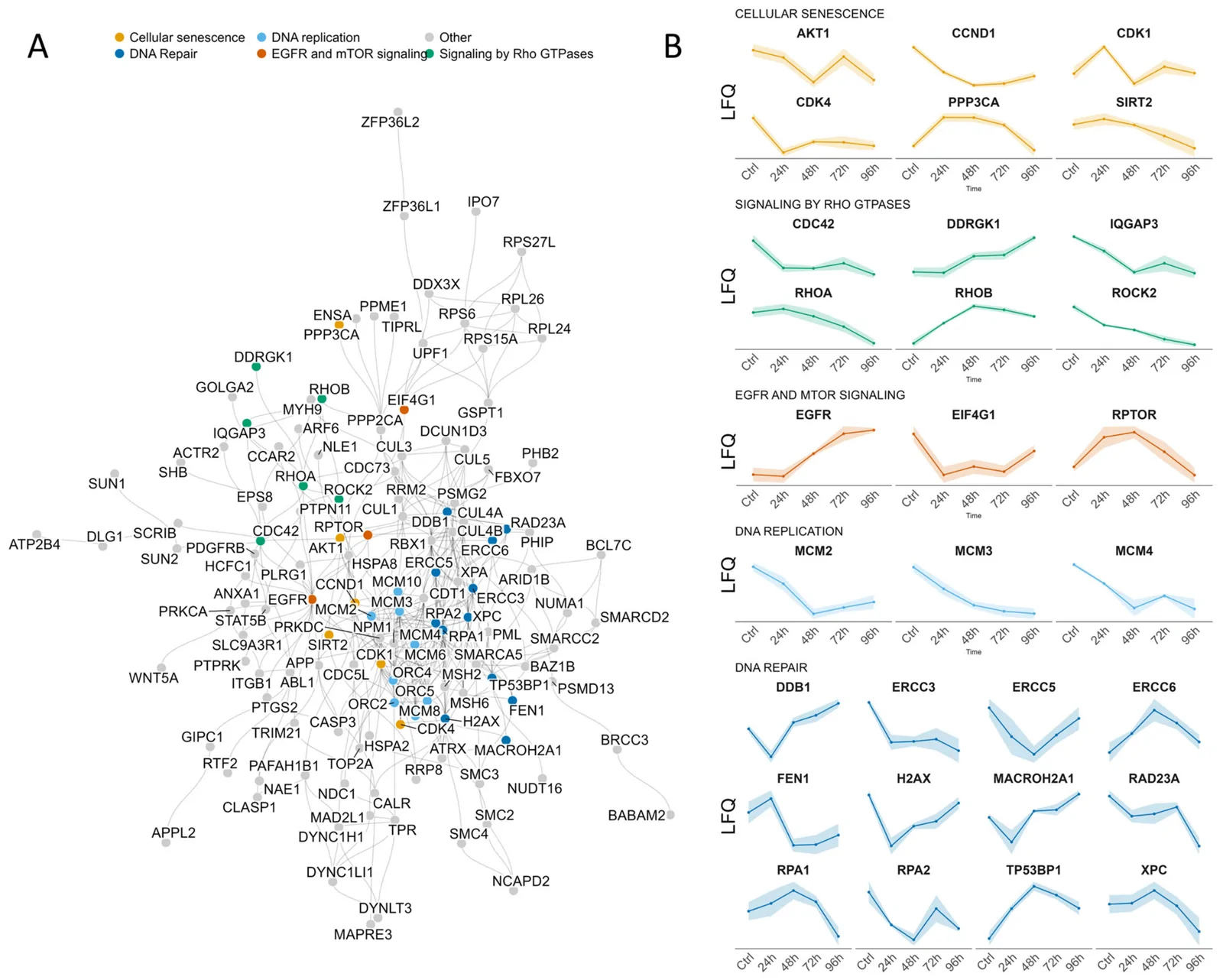
Chronic exposure to IL-1β (10 ng/mL) leads to coordinated abundance changes in proteins related to DNA maintenance, replication and repair signaling pathways in chondrocytes. (**A**) STRING protein–protein interaction network generated from proteins identified in significantly enriched Gene Ontology (GO) biological processes associated with cell cycle regulation. Proteins were selected from the list of differentially expressed proteins identified by FDR-corrected ANOVA and subsequently grouped according to enriched GO terms, including cellular senescence, DNA replication, DNA damage response and repair, EGFR and mTOR signaling, and Rho GTPase-mediated signaling. Nodes represent proteins and edges represent known or predicted interactions retrieved from the STRING database. (**B**) Representative temporal expression profiles of selected hub proteins are shown, illustrating coordinated regulation across the experimental time course. For all panels, protein abundance is expressed as mean log2 LFQ. “Ctrl” represents untreated chondrocytes cultured for 96 h under contact-inhibited conditions, which were used as the reference group to account for potential effects of culture duration, cell confluency, and cell-cycle status. Lines represent mean protein abundance values and shaded areas indicate standard deviation.

Since we observed down-regulation of DNA replication and translation-associated processes at later time points, we next assessed how proteins governing the cell cycle responded to sustained IL-1β treatment. To this end, significantly regulated proteins annotated with the Gene Ontology term “cell cycle” were retrieved and used to generate a STRING protein–protein interaction network (Figure 3A). The resulting network comprised proteins associated with DNA replication, DNA damage response, Rho GTPase signaling, cellular senescence, and EGFR/mTOR signaling. Representative temporal expression profiles of selected proteins from these functional categories are shown in Figure 3B.

Within this network, proteins associated with canonical cell cycle control and senescence exhibited distinct temporal regulation patterns. While CDK1 showed altered expression at 24 h post-treatment, its expression remained comparable between control and IL-1β-treated cells at 96 h. In contrast, CDK4 showed persistent down-regulation across all IL-1β-treated time points relative to control cells. CCND1 (Cyclin D1), which positively regulates CDK4/6-mediated phosphorylation of the retinoblastoma (Rb) protein and promotes progression from G1 to S phase [36], was also down-regulated at later time points. In parallel, several subunits of the minichromosome maintenance (MCM) complex were consistently down-regulated over time, further reflecting altered regulation of DNA replication-associated processes. These abundance measurements do not directly assess CDK4-Cyclin D1 kinase activity or Rb phosphorylation.

Additional proteins associated with growth factor signaling, DNA damage response, and chromatin organization also exhibited dynamic regulation throughout the experimental period (representative protein temporal profiles are also shown in Figure 3). EGFR expression increased following IL-1β stimulation; however, downstream components of the pathway, including AKT1 and RPTOR, displayed temporally heterogeneous responses rather than sustained up-regulation. Similarly, proteins involved in DNA maintenance and chromatin remodeling showed distinct temporal profiles. TP53BP1 was progressively upregulated at later time points, whereas H2AX, macroH2A1 exhibited more transient modulation, suggesting that prolonged IL-1β exposure engages DNA damage and chromatin-associated pathways in a temporally coordinated but non-uniform manner.

**Figure 4:**
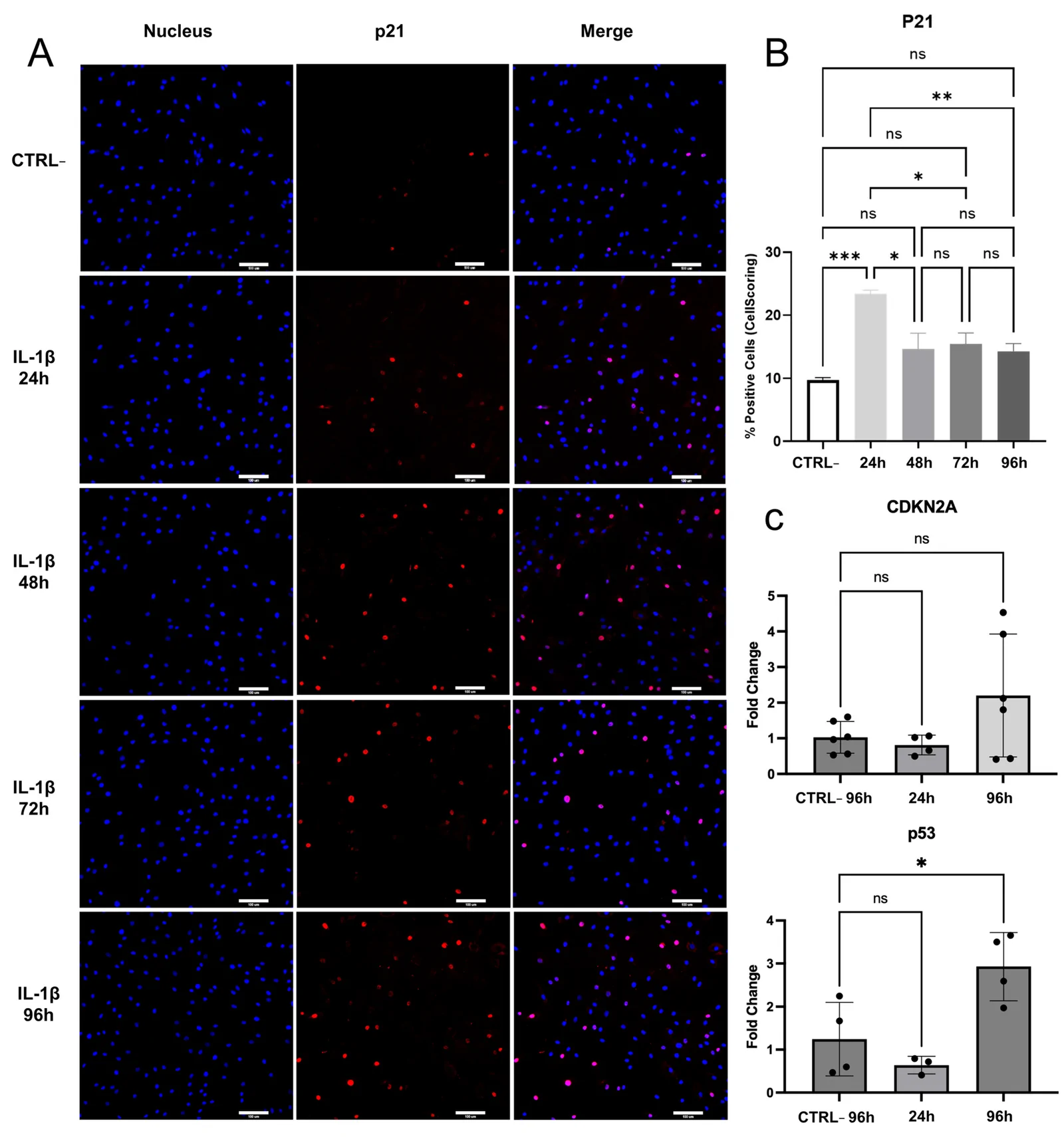
Dynamic modulation of senescence-associated markers (p21 and p53) during sustained IL-1β exposure. (**A**) Representative immunofluorescence images of p21 expression in chondrocytes exposed to sustained IL-1β stimulation. NHAC-Kn cells were stained for p21 (red) and nuclei (Hoechst; blue). Contact-inhibited, untreated early-passage chondrocytes cultured for 96 h (P3; CTL−) were included as reference conditions. Cells were exposed to IL-1β (10 ng/mL) for 24 h, 48 h, 72 h, and 96 h. Representative merged images show p21-positive cells. Scale bars = 100 μm. (**B**) The graph on the right shows the quantification of the percentage of p21-positive cells using HCS-based cell scoring analysis. Data are presented as mean ± SD from three independent experiments obtained from one donor. Statistical analysis was performed using GraphPad Prism 11. * *p* < 0.05; ** *p* < 0.01; ns, not significant. Scale bars: 100 µm. (**C**) qRT-PCR analysis of senescence-associated genes following sustained IL-1β exposure. Transcript levels of CDKN2A (p16^INK4a^) and TP53 (p53) were quantified by qRT-PCR in untreated early-passage chondrocytes (contact-inhibited, cultured for 96 h) and cells exposed to IL-1β (10 ng/mL) for 24 h and 96 h. Gene expression was normalized to endogenous reference genes (RPL37A and B2M) and is presented as fold change relative to the untreated control condition (*n* = 3 independent culture-level experiments from one donor, each performed with technical duplicates). Statistical significance was determined by one-way ANOVA followed by Tukey’s multiple-comparison test. * *p* < 0.05, ** *p* < 0.01, *** *p* < 0.001, and ns, not significant.

Given the sustained downregulation of CDK4 and Cyclin D1, we next examined the expression of upstream regulators associated with cell-cycle arrest during senescence. The activity of the CDK4–Cyclin D1 complex can be inhibited by upregulation of cyclin-dependent kinase inhibitors such as p21 [37], a key regulator of senescence [38]. Immunofluorescence analysis (shown in Figure 4A,B) revealed a transient increase in p21-positive cells following IL-1β exposure, with the strongest effect observed at 24 h. Notably, p21 induction was not sustained throughout the experimental period, whereas down-regulation of CDK4, Cyclin D1, and DNA replication-associated proteins persisted at later time points. These findings are consistent with an early association between p21 up-regulation and cell cycle arrest, followed by persistent abundance changes in CDK4, Cyclin D1, and DNA replication-associated proteins. Because kinase activity and Rb phosphorylation were not measured in this study, the data do not establish that these changes causally maintain growth arrest.

Additional senescence-associated markers displayed dynamic responses to sustained IL-1β stimulation. Proteomic analysis revealed modulation of the nuclear lamina protein LMNB1 during the early phase of treatment, whereas p16^INK4a^ did not reach statistical significance at any experimental time point (Supplementary Figure S1). Consistent with these observations, qRT-PCR analysis (Figure 4C) showed no significant changes in CDKN2A (p16^INK4a^) expression following 96 h of IL-1β exposure. In contrast, TP53 transcript levels were significantly increased following 96 h of IL-1β exposure. Together with the transient increase in p21-positive cells, these findings indicate that sustained IL-1β exposure is associated with dynamic changes in selected senescence-associated markers.

**Figure 5:**
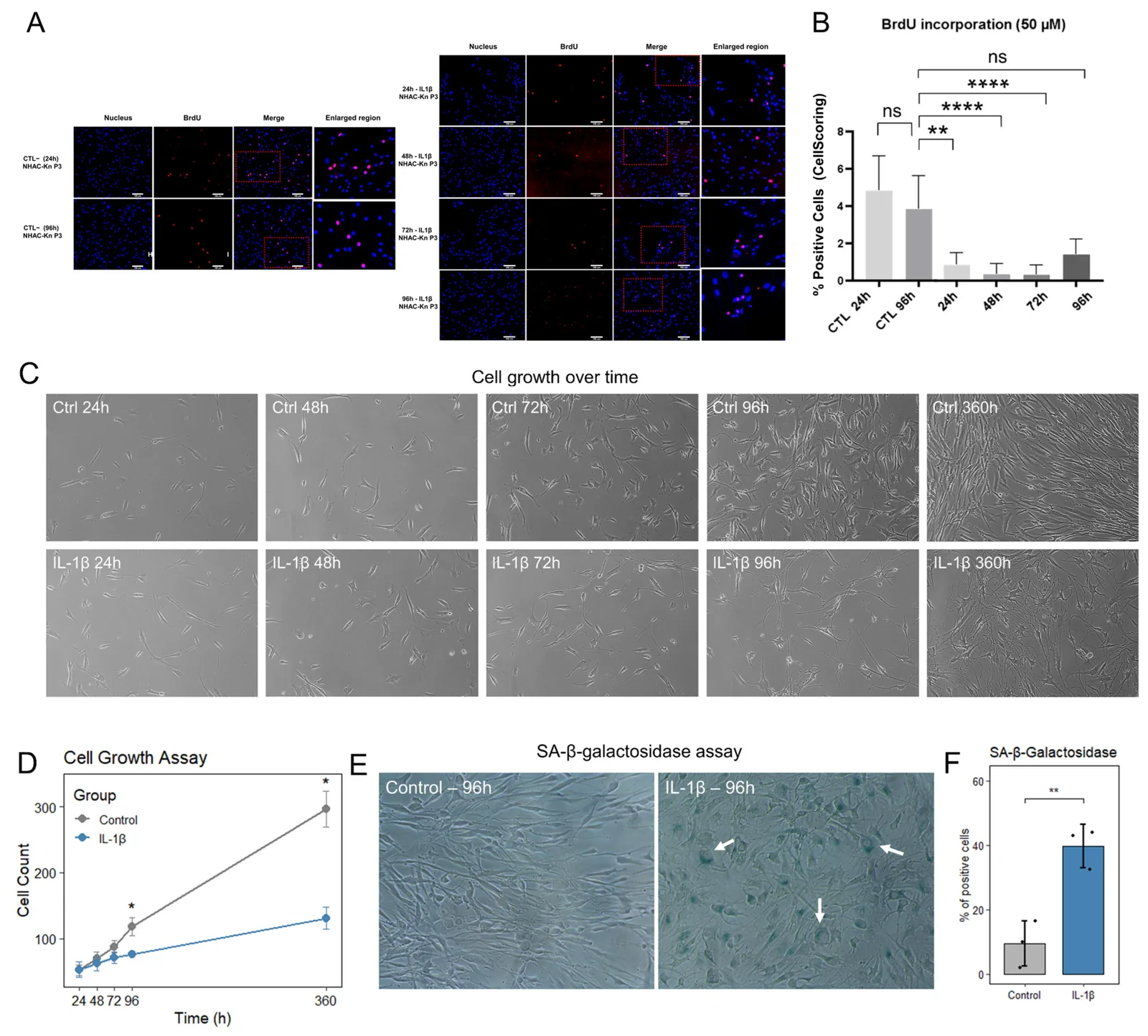
Sustained IL-1β exposure is associated with impaired chondrocyte proliferation and promotes senescence-associated traits, with partial recovery of BrdU incorporation at one post-withdrawal time point. (**A**) Representative immunofluorescence images of NHAC-kn cells subjected to BrdU incorporation assay and analyzed by High-Content Screening. Cells were pulsed with BrdU (50 µM) for 4 h before fixation, followed by DNA denaturation with HCl for 10 min, neutralization with sodium borate buffer for 5 min, and staining with anti-BrdU PerCP-Cy5.5. Nuclei were counterstained with Hoechst and are shown in blue, while BrdU-positive nuclei are shown in red. Images are arranged in four columns showing, from left to right, the nuclei, BrdU staining, the merged channels, and magnified views of selected regions. The experimental groups include untreated early-passage chondrocytes analyzed after 24 h (CTL− 24 h), untreated early-passage chondrocytes maintained under contact-inhibited conditions for 96 h (CTL− 96 h), and chondrocytes exposed to IL-1β (10 ng/mL) for 24 h, 48 h, 72 h, and a recovery condition consisting of 96 h of IL-1β exposure followed by 48 h in cytokine-free medium. The red dashed-boxes indicate the regions enlarged in the fourth column. (**B**) The graph shows the quantification of the percentage of BrdU-positive cells using HCS-based cell scoring analysis. Data are presented as mean ± SD from three independent experiments obtained from one donor. Statistical analysis was performed using GraphPad Prism 11. * *p* < 0.05; ** *p* < 0.01; **** *p* < 0.0001; ns, not significant. Scale bars: 100 µm. (**C**) Representative phase-contrast images of chondrocytes cultured under control conditions or treated with IL-1β (10 ng/mL) for 24 h, 48 h, 72 h, 96 h, and 360 h are shown. (**D**) Cell growth was quantified by cell counting over time, with cell numbers expressed as ×10^3^ cells (*n* = 3 independent experiment from 1 donor). (**E**) SA-β-gal staining was performed at 96 h, with representative images shown for control and IL-1β–treated cells (white arrows indicate SA-β-gal-positive cells displaying morphological alterations, including cell body and nuclei enlargement), together with quantification of SA-β-gal-positive cells. Statistical significance was determined by Student’s T test (**F**) (*n* = 3 independent experiment from 1 donor). * *p* < 0.05, ** *p* < 0.01, and **** *p* < 0.0001; ns, not significant.

To determine whether these molecular alterations translated into functional impairment of cell-cycle progression, DNA synthesis was assessed by BrdU incorporation (Figure 5A,B). To account for potential effects of cell density and confluence on proliferative activity, early-passage untreated chondrocytes cultured for 24 h and 96 h were included as independent negative controls. Sustained IL-1β exposure significantly reduced the proportion of BrdU-positive cells throughout the experimental period, indicating suppression of proliferative activity (Figure 5A,B). At a single post-withdrawal time point assessed, chondrocytes showed partial recovery of BrdU incorporation; however, values remained below those of proliferating control cultures. This result indicates limited recovery of DNA synthesis under the tested conditions but does not establish reversibility of the broader cellular phenotype.

Consistent with these findings, long-term growth assays demonstrated progressive impairment of cell expansion under continuous IL-1β stimulation (Figure 5C,D). Significant differences in cell number emerged after 96 h of treatment and became more pronounced during extended culture, remaining evident after 360 h of continuous exposure. These observations indicate that sustained IL-1β signaling not only suppresses DNA synthesis but also compromises the long-term proliferative capacity of chondrocytes, which could be relevant under contexts of chronic inflammation.

Given the observed proliferative arrest, we next investigated whether prolonged IL-1β exposure was accompanied by the acquisition of other senescence-associated features. Senescence-associated β-galactosidase (SA-β-gal) activity was significantly increased following 96 h of IL-1β treatment compared with control cells (Figure 5E,F) and was interpreted alongside the other molecular and cellular findings, as SA-β-gal activity alone is insufficient to establish cellular senescence. In addition to enhanced SA-β-gal staining, several IL-1β-treated chondrocytes displayed morphological features commonly associated with senescence, including enlarged cell bodies and nuclei (shown by the white arrows in Figure 5E).

Consistent with these phenotypic alterations, proteomic analyses revealed sustained abundance remodeling of proteins involved in cytoskeletal regulation and growth factor-associated signaling. Several proteins associated with the Rho GTPase network, including RHOA, CDC42, and ROCK2, were downregulated at later timepoints, especially 96 h post-treatment. These proteins play important roles in cytoskeletal organization, mechanotransduction, and cell-cycle progression [39,40]. In contrast, RHOB displayed transient upregulation during the early stages of IL-1β exposure before stabilizing at later time points. These measurements reflect protein abundance and do not directly establish altered Rho GTPase activity.

**Figure 6:**
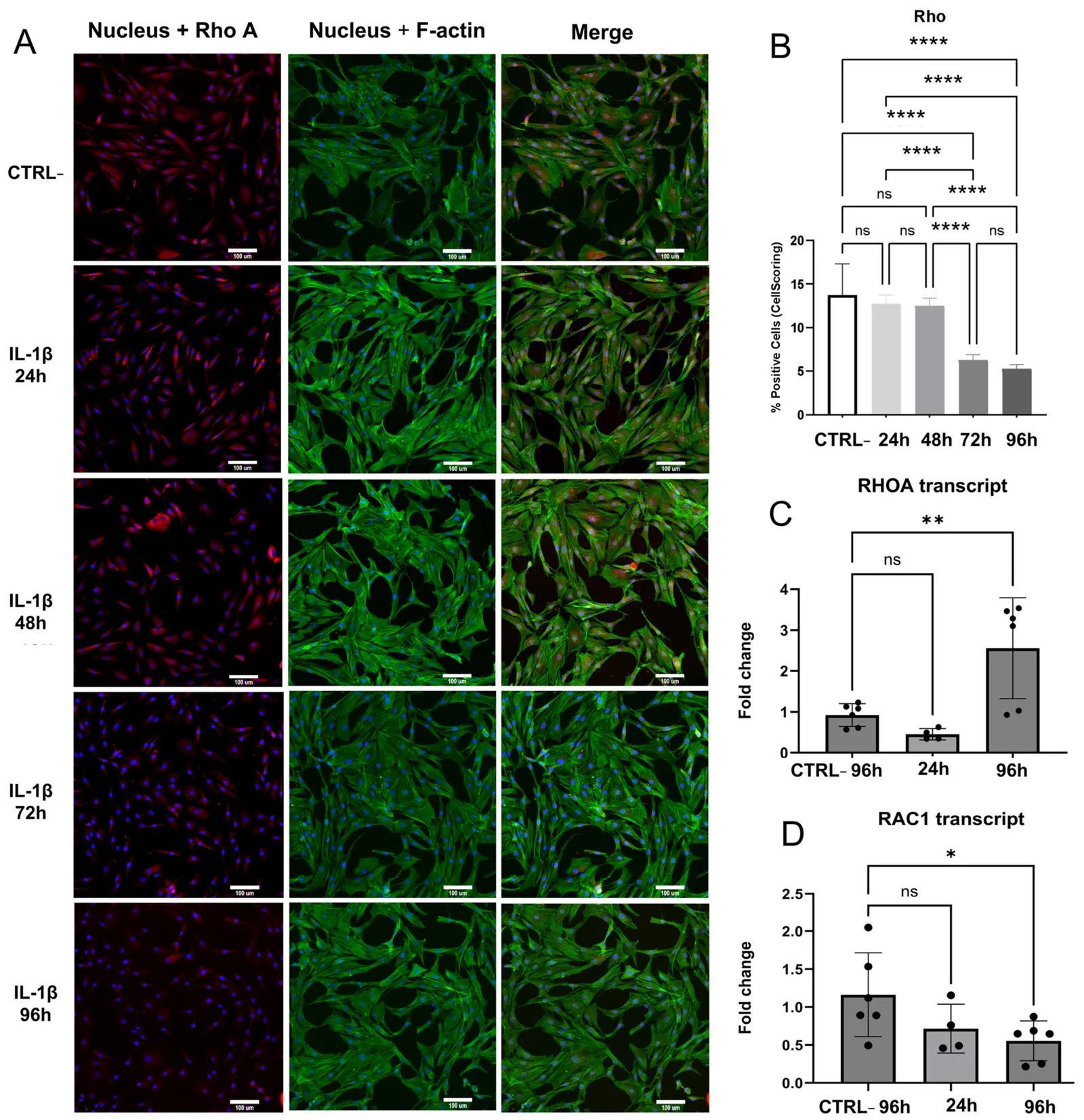
Long-term down-regulation of RHOA abundance and remodeling in chondrocytes exposed to IL-1β. (**A**) Representative immunofluorescence images of NHAC-Kn cells stained for RHOA (red), F-actin (Phalloidin-488; green), and nuclei (Hoechst; blue). Experimental groups include untreated early-passage chondrocytes cultured for 96 h (P3; CTL−), and P3 chondrocytes exposed to IL-1β (10 ng/mL) for 24 h, 48 h, 72 h, or 96 h. Scale bars = 100 μm. (**B**) Quantification of RHOA-positive cells determined by high-content screening-based cell scoring analysis. Data are presented as mean ± SD from three independent experiments derived from 1 donor. Statistical significance was determined by one-way ANOVA followed by Tukey’s multiple-comparison test. * *p* < 0.05; ** *p* < 0.01; **** *p* < 0.0001; ns, not significant. (**C**,**D**) qRT-PCR analysis of RHOA and RAC1 transcript levels, respectively, following IL-1β treatment for 24 and 96 h. Gene expression was normalized to endogenous reference genes (RPL37A and B2M) and is presented as fold change relative to the untreated control condition (*n* = 3 independent biological experiments derived from 1 donor, each performed in technical duplicate). Statistical significance was determined by one-way ANOVA followed by Tukey’s multiple-comparison test. * *p* < 0.05, ** *p* < 0.01, and **** *p* < 0.0001; ns, not significant.

To corroborate these findings, RHOA abundance was assessed by immunofluorescence (Figure 6A,B) in one of the same donors assessed in the proteomics experiment. Consistent with the proteomic data, sustained IL-1β exposure resulted in a progressive reduction in RHOA protein abundance, with the most pronounced decrease observed at 72–96 h. To compare transcript and protein abundance patterns for Rho GTPase-associated genes, RHOA and RAC1 transcript levels were quantified by qRT-PCR (Figure 6C,D). Interestingly, the transcriptional profiles differed from the protein-level observations. Whereas RHOA mRNA expression was significantly increased after 96 h of IL-1β exposure, RAC1 transcripts were significantly reduced at the same time point. We hypothesize that increased RHOA transcript levels despite reduced protein abundance may reflect post-transcriptional regulation mechanisms. In contrast, the downregulation of RAC1 is consistent with the often antagonistic roles of RHOA and RAC1 in regulating cytoskeletal organization [41].

**Figure 7:**
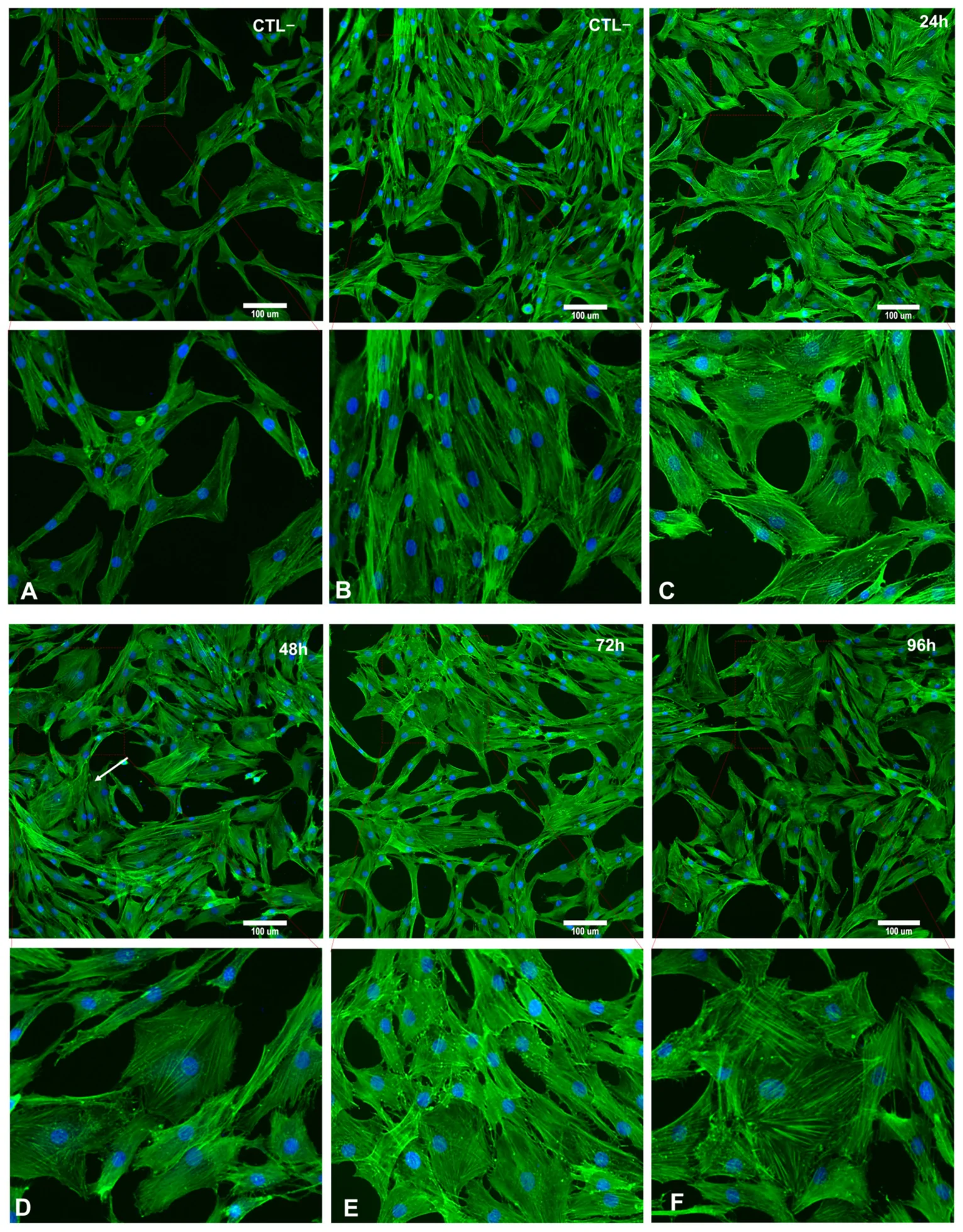
Abundance changes in Rho GTPase-associated proteins is accompanied by changes in the actin cytoskeleton organization and cell morphology. Representative immunofluorescence images of NHAC-kn cells stained for F-actin with Phalloidin-488 (green) and nuclei with Hoechst (blue). For each condition, the upper panels show representative overview images, while the lower panels show magnified regions selected by the red dashed boxes. (**A**,**B**) Representative images of untreated early-passage chondrocytes cultured for 96 h (CTL−). (**C**–**F**) Time-course analysis of NHAC-kn cells chronically exposed to 10 ng/mL IL-1β for 24 h (**C**), 48 h (**D**), 72 h (**E**), and 96 h (**F**). Arrows indicate representative actin filament bundles. Scale bars = 100 μm. Three independent experiments derived from 1 donor were performed.

In parallel, qualitative confocal microscopy analysis of F-actin staining revealed a time-dependent cytoskeletal remodeling in NHAC-kn cells chronically exposed to IL-1β, as shown in Figure 7. Compared with untreated controls, which displayed a small, elongated, spindle-shaped morphology consistent with a young, proliferative phenotype, IL-1β-treated cells progressively acquired an enlarged, flattened appearance, consistent with the morphological changes observed in the SA-β-gal assay. These changes were already evident at 24 h and 48 h and became more pronounced at 72 h and 96 h, when cells exhibited increased F-actin fluorescence intensity and prominent stress-fiber-like actin structures, suggesting progressive cytoskeletal stress and altered actin polymerization during chronic inflammation-induced stress. Together, these findings demonstrate that sustained IL-1β exposure was associated with a growth-arrested state characterized by impaired proliferation, cytoskeletal remodeling, and the dynamic, time-dependent regulation of several senescence-associated traits. Nevertheless, post-withdrawal BrdU measurement showed partial recovery of DNA synthesis at a single time-point, suggesting that this inflammation-induced state retains some degree of proliferative plasticity and may not correspond to a fully irreversible senescence state, even though we have not assessed the reversibility of the broader cellular phenotype.

### 3.3. Sustained IL-1β Exposure Induces Coordinated Abundance Remodeling of Organelle Homeostasis-Associated Proteins and Metalloprotease in Conditioned Mediums

Given the early enrichment of oxidative stress-related processes and the emergence of early signs of senescence, we next examined whether sustained IL-1β exposure induces metabolic and organelle-level adaptations consistent with chronic oxidative stress. To address this, we analyzed the set of proteins exhibiting significant temporal regulation by mapping them to curated organelle-associated sub-processes, enabling a systematic assessment of mitochondrial, ER, Golgi, and lysosomal proteomic adaptations over time.

**Figure 8:**
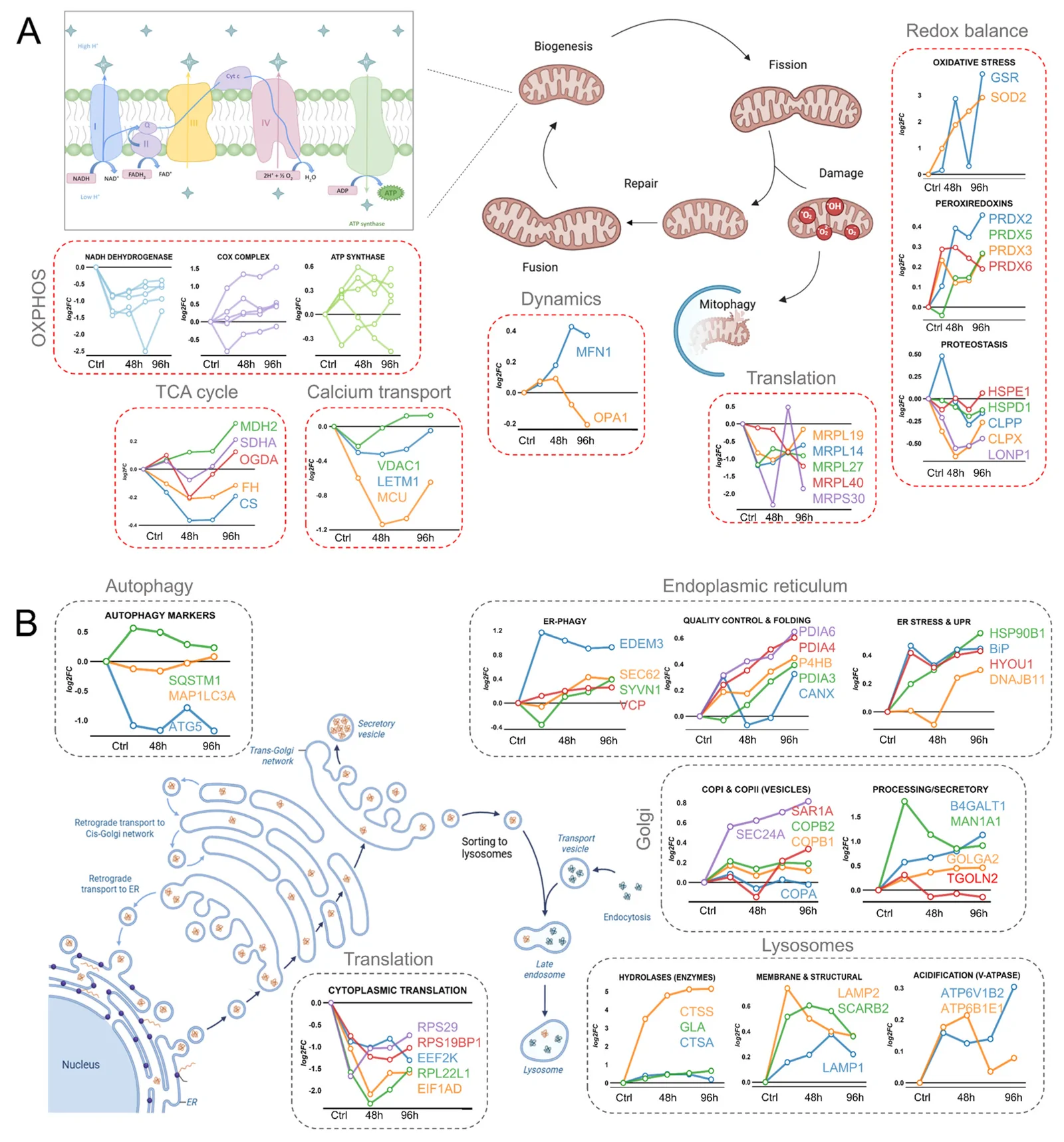
Temporal regulation of proteins involved in mitochondrial stress responses, ER stress, Golgi processing, and lysosome-mediated quality control in chondrocytes exposed to IL-1β (10 ng/mL) for up to 96 h. (**A**) Schematic representation of mitochondrial-associated protein groups overlaid with temporal proteomic data. Line plots show mean log_2_ fold-change values for representative proteins associated with oxidative phosphorylation complexes, mitochondrial ribosomal proteins, redox-associated proteins, and mitochondrial proteases. (**B**) Temporal expression profiles of proteins associated with the endoplasmic reticulum (ER), Golgi apparatus, and lysosomes. Protein groups include ER-associated folding proteins, unfolded protein response (UPR)-related proteins, ER-phagy-associated proteins, COPI/COPII vesicle trafficking proteins, Golgi-associated proteins, lysosomal hydrolases, lysosomal membrane proteins, and V-ATPase subunits. For all panels, protein abundance is expressed as log_2_ fold change relative to untreated chondrocytes cultured for 96 h under contact-inhibited conditions, which were used as the reference group to account for potential effects of culture duration, cell confluency, and cell-cycle status. The “Ctrl” value corresponds to the reference control condition relative to itself (log_2_ fold change = 0). Lines represent mean protein abundance values.

Regarding mitochondrial metabolism, OXPHOS components displayed heterogeneous temporal responses, with sustained downregulation of Complex I subunits concomitant with progressive upregulation of Complex IV and ATP synthase components (representative profile plots for these proteins are shown in Figure 8A). Prolonged IL-1β exposure was also associated with increased abundance of several antioxidant and redox-regulating proteins. Key redox-regulating enzymes, including SOD2, GSR, and multiple peroxiredoxins, were robustly upregulated over time. In contrast, several proteins associated with mitochondrial maintenance and proteostasis exhibited a general decline. Proteases and chaperones such as LONP1 showed reduced expression, accompanied by downregulation of mitochondrial ribosomal proteins. Additionally, altered expression of mitochondrial fusion regulators, such as MFN1 and OPA1, was also observed.

As shown in Figure 8B, proteins involved in ER protein folding and quality control were progressively upregulated, including multiple protein disulfide isomerases and ER-resident chaperones. This was accompanied by increased abundance of unfolded protein response-associated factors, consistent with remodeling of ER stress-response pathways. In parallel, components associated with ER-phagy pathways were up-regulated at later time points, indicating remodeling of proteins linked to selective autophagy. Analysis of autophagy-related markers revealed a trend toward increased SQSTM1 expression together with reduced ATG5 levels. Notably, cytoplasmic ribosomal proteins and some translation initiation factors were downregulated, indicating remodeling of the protein-synthesis machinery, a hallmark of chronic stress phenotypes [42].

Within the Golgi and vesicular trafficking pathways, a more pronounced upregulation of the COPII component SEC24 compared to COPI subunits such as COPA and COPB indicates differential remodeling of proteins associated with anterograde ER-to-Golgi and retrograde Golgi-to-ER trafficking. In parallel, the sustained upregulation of some glycosylation enzymes together with the Golgi structural protein GOLGA2 indicates increased abundance of proteins associated with Golgi organization and protein processing. Notably, we noticed a subtle downregulation of the trans-Golgi network export marker TGOLN2, involved in post-Golgi trafficking dynamics. Concomitantly, lysosomal proteome remodeling was evident through increased abundance of lysosomal hydrolases, membrane proteins, and V-ATPase subunits. These alterations align with the increased SA-β-galactosidase activity observed in IL-1β-treated chondrocytes.

**Figure 9:**
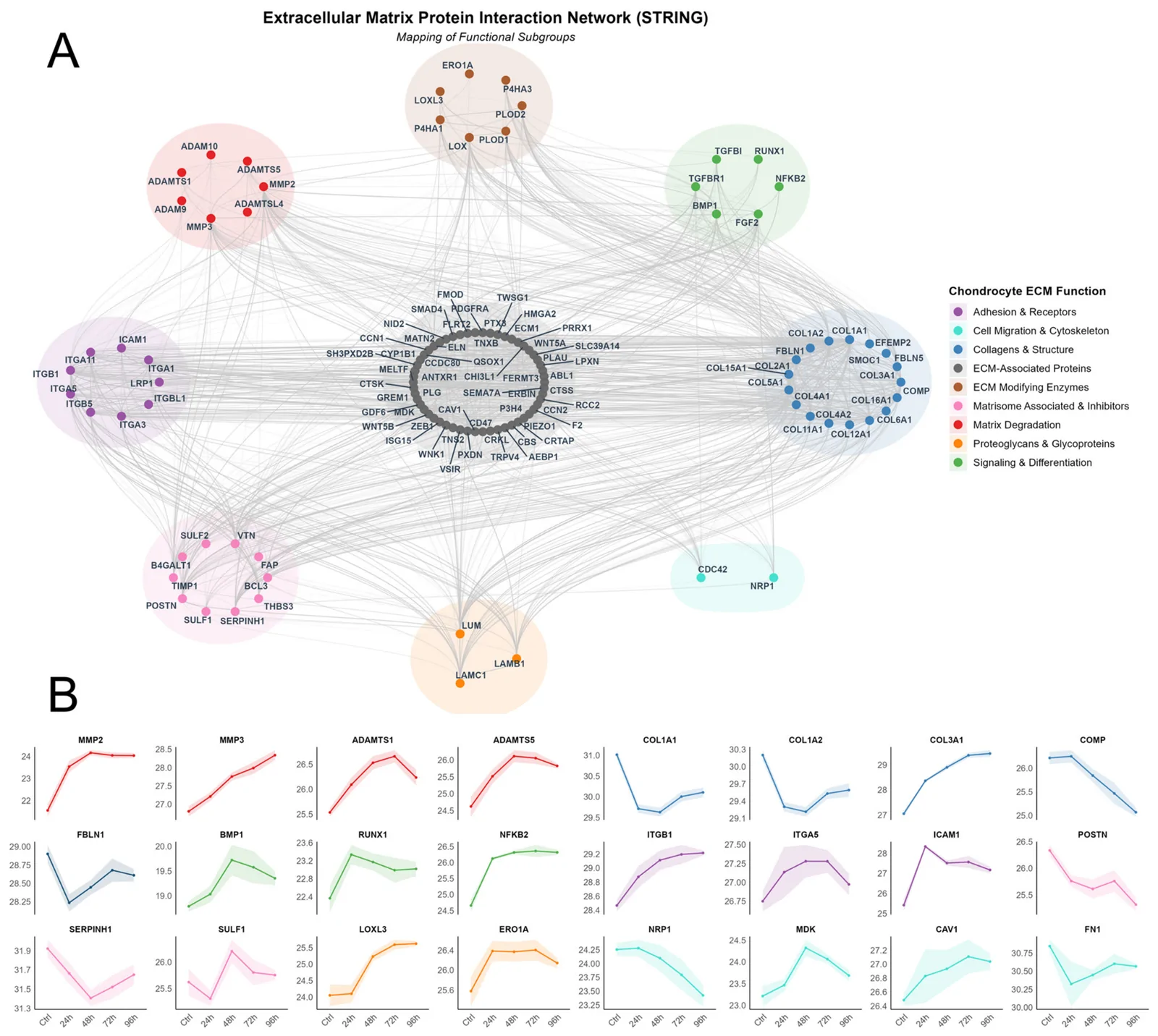
Abundance intracellular remodeling of ECM-associated proteins during sustained IL-1β exposure is characterized by reduced structural matrix proteins and increased intracellular catabolic mediators. (**A**) STRING protein–protein interaction network generated from differentially abundant ECM-associated proteins identified in IL-1β-treated chondrocytes. The network was generated using significantly regulated ECM-associated proteins identified by FDR-corrected ANOVA. Proteins were manually grouped according to their annotated biological functions, including collagens and structural ECM components, matrix degradation, ECM-modifying enzymes, adhesion and receptor proteins, cell migration and cytoskeleton-associated proteins, matricellular proteins and inhibitors, proteoglycans and glycoproteins, and signaling and differentiation-related proteins. Nodes represent individual proteins and edges represent known or predicted protein–protein interactions retrieved from the STRING database. (**B**) Representative temporal expression profiles of selected proteins from the ECM-associated network across the experimental time course. Mean expression values are shown for control cells and cells exposed to IL-1β for 24 h, 48 h, 72 h, and 96 h. The control condition was maintained for the same duration as the longest treatment group (96 h) to minimize differences related to culture time, confluency, and cell-cycle progression. Shaded areas represent the standard deviation associated with each measurement. Proteins displayed include matrix-degrading enzymes (MMP2, MMP3, ADAMTS1, and ADAMTS5), structural ECM proteins (COL1A1, COL1A2, COL3A1, COMP, and FBLN1), signaling and differentiation-associated proteins (BMP1, RUNX1, and NFKB2), adhesion-related proteins (ITGB1, ITGA5, and ICAM1), matricellular proteins and ECM regulators (POSTN, SERPINH1, and SULF1), ECM-modifying enzymes (LOXL3 and ERO1A), and additional ECM-associated proteins (NRP1, MDK, CAV1, and FN1).

Parallel to these proteomic adaptations, prolonged exposure to IL-1β induced a coordinated remodeling in the intracellular abundance of ECM-associated proteins identified through GO enrichment. Analysis of the STRING protein interaction network (shown in Figure 9A) revealed temporal reprogramming across distinct functional subgroups of ECM-related proteins, characterized by the intracellular downregulation of core structural components and the simultaneous upregulation of degradative machinery and inflammatory signaling. As shown in Figure 9A,B, at the core of the structural alterations, a progressive intracellular downregulation was observed for fibrillar and non-fibrillar collagens essential for cartilage tissue homeostasis, including COL2A1 (temporal profile for this protein is shown in Supplementary Figure S2), the principal collagen of articular cartilage, together with COL1A1, COL1A2, and COL3A1, as well as matrix scaffolding and organizing proteins such as cartilage oligomeric matrix protein (COMP) and fibulin-1 (FBLN1). This loss of structural components was accompanied by reduced intracellular abundance of proteins involved in collagen maturation and extracellular matrix organization, including SERPINH1 (HSP47), a collagen-specific molecular chaperone, and sulfatase 1 (SULF1), an extracellular sulfatase that modulates growth factor signaling through heparan sulfate remodeling. Conversely, chondrocytes exhibited a delayed response characterized by increased intracellular abundance of proteins associated with extracellular matrix remodeling and cellular stress adaptation, including lysyl oxidase-like 3 (LOXL3) and ERO1A, a regulator of oxidative protein folding and redox homeostasis.

Consistent with this intracellular remodeling profile of ECM-associated proteins, temporal analysis (shown in Figure 9B) highlighted an intracellular increase in proteins associated with matrix degradation and inflammatory signaling, as would be expected of cells stimulated with IL-1β. Key proteolytic enzymes, including matrix metalloproteinases MMP2 and MMP3, alongside the aggrecanases ADAMTS1 and ADAMTS5, displayed sustained intracellular up-regulation over the 96 h course. This phenotype was accompanied by increased abundance of signaling and regulatory proteins, including NFKB2 and RUNX1, as well as cell adhesion receptors and molecules including ITGB1, ITGA5, and ICAM1. Notably, integrins such as ITGB1 and ITGA5 may contribute to the observed phenotype, as integrin-mediated signaling has been shown to regulate chondrocyte responses to extracellular matrix cues and to modulate matrix metalloproteinase (MMP) expression [43,44].

**Figure 10:**
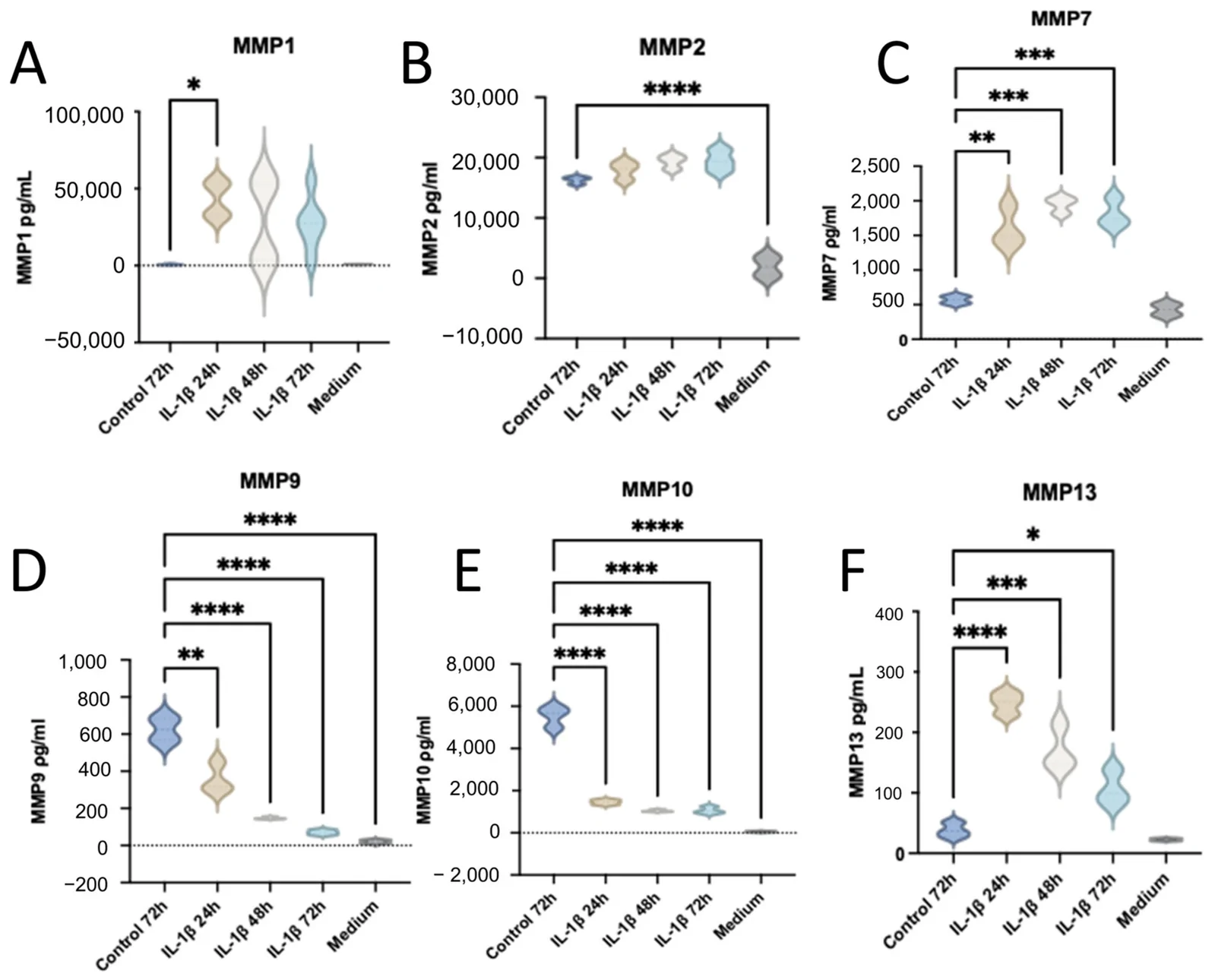
Temporal metalloproteinase secretion profiles mirror proteomic changes observed during sustained IL-1β exposure. (**A**) MMP1, (**B**) MMP2, (**C**) MMP7, (**D**) MMP9, (**E**) MMP10, and (**F**) MMP13 concentrations were measured in culture supernatants collected from chondrocytes exposed to IL-1β (10 ng/mL) for 24 h, 48 h, and 72 h. Untreated cells cultured for 72 h were included as controls. Medium-only samples were included as negative controls to assess background signal in the absence of cells. Metalloproteinase concentrations are presented as pg/mL. Violin plots show the distribution of individual measurements. Statistical significance was determined by one-way ANOVA followed by Tukey’s multiple-comparison test (*n* = 3 independent experiments derived from 1 donor). * *p* < 0.05, ** *p* < 0.01, *** *p* < 0.001, and **** *p* < 0.0001.

To determine whether these proteomic alterations were accompanied by changes in the extracellular release profile, cytokines and matrix metalloproteinases were quantified in conditioned media. Multiplex cytokine profiling performed after 96 h of treatment revealed an inflammatory conditioned-medium profile characterized by higher concentrations of IL-6 and TNFα than in untreated controls (Supplementary Figure S3). These findings were accompanied by marked changes in the conditioned-medium concentrations of multiple metalloproteinases (results shown in Figure 10). MMP1 and MMP13 concentrations were highest after 24 h of IL-1β exposure (Figure 10A,F). By contrast, MMP2 displayed a progressive upward trend that mirrored the intracellular proteomic profile, although this increase did not reach statistical significance (Figure 10B). Conversely, MMP7 concentrations increased progressively across the experimental time points (Figure 10C). Interestingly, MMP9 and MMP10 concentrations progressively decreased throughout the experimental period (Figure 10D,E). Because the culture medium was replaced daily, these measurements reflect the release of each factor during the interval preceding sample collection rather than cumulative accumulation throughout the experimental period. Overall, these temporal patterns indicate exposure-duration-associated remodeling of the MMP release profile during sustained IL-1β stimulation.

## 4. Discussion

Although IL-1β has been extensively used as a model of inflammatory stress in chondrocytes, most studies have focused on acute responses [9,10,13,21,22,23], leaving the molecular consequences of sustained inflammatory signaling poorly characterized. By combining time-resolved DIA proteomics with complementary molecular and cellular assays, we demonstrate that prolonged IL-1β exposure induces extensive remodeling of pathways governing extracellular matrix homeostasis, cell-cycle progression, cytoskeletal organization, mitochondrial function, and organelle proteostasis. These molecular alterations are accompanied by impaired proliferative capacity and the emergence of transient senescence-associated traits.

Among the molecular changes induced by sustained IL-1β exposure, abundance remodeling of cell-cycle regulatory proteins emerged as a key feature associated with the observed growth-arrest phenotype. Network analysis revealed coordinated downregulation of CDK4, Cyclin D1, and multiple components of the minichromosome maintenance (MCM) complex. Because CDK4-Cyclin D1 activity is required for phosphorylation of the retinoblastoma protein and progression through the G1/S restriction point [36,45], sustained downregulation of components of this pathway is consistent with impaired cell-cycle entry and reduced proliferative competence. Similarly, the MCM complex functions as the replicative helicase required for DNA replication initiation and S-phase progression [46], further supporting the notion that chronic IL-1β signaling compromises DNA replication capacity. BrdU incorporation assays corroborated these proteomic findings, as IL-1β-treated chondrocytes exhibited reduced BrdU incorporation and progressive impairment of long-term cell proliferation. However, CDK4-Cyclin D1 activity, Rb phosphorylation, and the causal contribution of this axis to the growth arrest were not directly tested in this study and remain to be investigated.

Interestingly, several canonical senescence-associated regulators, such as p21 and LMNB1 [38,47,48], displayed transient responses. The expression of p21 increased only during the early phase of IL-1β exposure. Also, LMNB1 was significantly altered only at early time points. These findings suggest that sustained inflammatory signaling regulates components of senescence-associated regulatory networks but does not maintain all classical senescence markers throughout the experimental period. Consistent with this interpretation, at a single post-withdrawal time point, chondrocytes showed partial recovery of BrdU incorporation. Because recovery was assessed only once and using a proliferation-based readout, these data indicated some degree of proliferative plasticity under the tested conditions but do not establish reversibility of the broader cellular phenotype. Together, these observations support the idea that prolonged inflammatory stimulation promotes a cellular state that shares important transient features with senescence while remaining distinct from fully established irreversible senescence programs.

Beyond cell-cycle regulation, chronic IL-1β was associated with abundance changes in proteins associated with cytoskeletal signaling pathways. Multiple components of the Rho GTPase network, including RHOA, CDC42, and ROCK2, were progressively downregulated particularly at later timepoints, and RHOA in particular was further assessed by immunofluorescence analyses using one of the donors evaluated in the proteomics experiment. These proteins are central regulators of cytoskeletal organization, mechanotransduction, cell migration, and also cell-cycle progression [39,40,49]. qRT-PCR revealed distinct transcriptional regulation of RHOA and RAC1 compared with the proteomic findings, which we hypothesize may involve post-transcriptional regulatory mechanisms, as has been previously reported in other contexts [50,51]. RHOA expression can be regulated directly at the translational level, as RNA-binding proteins such as hnRNP-Q1 have been shown to bind RHOA mRNA and repress its translation [50]. In addition, components of the Rho GTPase pathway are subject to transcriptional regulation and feedback mechanisms [51]. Thus, the late increase in RHOA transcripts may represent a compensatory transcriptional response that was not accompanied by a corresponding increase in protein synthesis. Although these mechanisms provide plausible explanations for the observed discordance, they were not directly investigated in this study. Because GTPase activation states and downstream effector activity were not measured, these findings should be interpreted as correlative abundance changes rather than direct evidence of altered Rho GTPase signaling activity.

Whereas RHOA transcripts were increased after prolonged IL-1β exposure despite reduced protein abundance, RAC1 expression was significantly decreased at 96 h, consistent with the often antagonistic roles reported for RHOA and RAC1 in cytoskeletal regulation [41]. Following these molecular alterations, chronic IL-1β exposure induced marked remodeling of the actin cytoskeleton, characterized by progressive cell enlargement, increased F-actin staining intensity, and the formation of prominent stress fiber-like actin structures. Such cytoskeletal changes are frequently associated with altered mechanotransduction, increased stress, and the acquisition of senescence-associated cellular features, impacting chondrocyte function [52,53,54].

In addition to their roles in cytoskeletal regulation, Rho GTPase activity has been shown to be required for quiescent cells to re-enter the cell cycle and initiate DNA synthesis [55,56]. Therefore, we hypothesize that downregulation of components of this signaling network may contribute to some degree to the impaired proliferative capacity observed in IL-1β-treated chondrocytes. In contrast, RHOB exhibited transient upregulation during the early stages of treatment. RHOB has been associated with stress responses, growth arrest, and p21-mediated signaling [57,58,59], suggesting that distinct members of the Rho family may play divergent roles during adaptation to chronic inflammatory stress. Interestingly, recent evidence has also described non-canonical intracellular functions of IL-1β involving interaction with the scaffold protein RACK1 and modulation of RhoA-dependent signaling pathways [60], raising a potential mechanistic hypohesis between inflammatory stimulation and the cytoskeletal remodeling observed in this study that will require direct functional testing.

Sustained IL-1β exposure also induced remodeling of several intracellular ECM-associated proteins. Progressive intracellular downregulation of COL2A1, the principal collagen of articular cartilage, together with reductions in COMP, FBLN1, and multiple collagen-processing proteins, are consistent with altered regulation of biological processes involving cartilage extracellular matrix maintenance, as previously reported [61,62]. However, because these measurements were obtained from the intracellular proteome, they do not directly demonstrate impaired matrix secretion, deposition, or integrity. These alterations were accompanied by increased intracellular abundance of canonical matrix-remodeling enzymes, including MMP2, MMP3, ADAMTS1, and ADAMTS5, as well as inflammatory mediators and adhesion-related proteins. Notably, however, targeted measurements of selected MMPs in conditioned media showed distinct temporal patterns, suggesting that extracellular protease release varied over the course of IL-1β exposure. Although the concentrations of some MMPs in the conditioned medium increased across IL-1β exposure durations, MMP9 and MMP10 concentrations progressively decreased. We hypothesize that chondrocytes may undergo a compensatory modulation in response to sustained cytokine exposure, potentially shifting their secretory phenotype to mitigate excessive proteolysis during prolonged inflammation under the specific IL-1β concentration and exposure regimen used in this study. Previous omics studies of IL-1β-treated chondrocytes have primarily characterized acute inflammatory responses and matrix-degrading programs [63,64]. Our findings extend these observations by suggesting that remodeling of the intracellular abundance of ECM-associated proteins persists alongside progressive alterations in cell-cycle, cytoskeletal, and organelle-associated proteomes during sustained cytokine exposure.

Furthermore, the coordinated abundance changes in intracellular matrix components and adhesion-associated proteins are consistent with remodeling of pathways involved in cell–matrix interactions, a process in which integrins serve as principal mechanosensors linking extracellular matrix composition to intracellular signaling outputs [64]. In chondrocytes, integrin-dependent signaling has been shown to modulate matrix-degrading responses and to cooperate with inflammatory cues in regulating metalloproteinase expression [43,44]. Importantly, altered integrin signaling has been shown in the literature to actively regulate the establishment and maintenance of cellular senescence phenotypes, with perturbations in integrin expression or signaling contributing to senescence induction and persistence [65,66].

Several aspects of the intracellular remodeling observed here are consistent with previous proteomic and functional studies describing mitochondrial dysfunction, oxidative stress, nitric oxide production [67,68], and endoplasmic reticulum stress [21] in IL-1β-treated chondrocytes. Nevertheless, earlier studies were generally limited to the analysis of few molecular markers, providing only a partial view of these processes [21,22,23]. The time-resolved proteomic approach employed here enabled simultaneous characterization of hundreds of proteins linked to mitochondrial biology, proteostasis, lysosomal function, and intracellular stress responses. In particular, we identified coordinated downregulation of multiple Complex I subunits alongside increased expression of downstream oxidative phosphorylation components, consistent with remodeling of mitochondrial energy metabolism previously reported [69]. These alterations were accompanied by changes in proteins governing mitochondrial proteostasis, including LONP1 and components of the mitochondrial translation machinery, indicating that chronic inflammatory signaling may affect not only respiratory function but also mitochondrial quality-control pathways, even though future studies are required to functionally validate those mechanisms.

In parallel, prolonged IL-1β exposure induced remodeling of the ER-Golgi-lysosome proteome axis. Proteins involved in protein folding, Golgi processing, vesicular trafficking, and lysosomal function were significantly altered throughout the experimental period. We hypothesize that such changes may reflect adaptive responses aimed at maintaining proteostasis under persistent inflammatory stress. These findings are consistent with a previous work from our group demonstrating induction of endoplasmic reticulum stress and alterations in secretion and glycosylation-related processes in chondrocytes exposed to hydrogen peroxide [25]. However, in that context mitochondrial alterations were not observed, suggesting that distinctive reactive species or redox mechanisms may be engaged depending on the nature of the stressor. Inflammatory stimulation with IL-1β has been reported to promote the production of multiple reactive oxygen and nitrogen species, including superoxide anions and nitric oxide [70,71,72,73], which together may influence mitochondrial function and contribute to the organelle-level remodeling observed here. Notably, alterations in mitochondrial and lysosomal proteomes, together with disruption of proteostasis-related pathways, are recurrent features of senescent cells [73,74,75]. The coordinated remodeling of these systems therefore provides additional molecular evidence that prolonged IL-1β exposure engages partial and predominantly transient cellular programs that overlap with senescence-associated states.

This study has several limitations. First, the experimental model relies on primary chondrocytes exposed to sustained IL-1β stimulation in vitro and therefore does not fully recapitulate the complex mechanical, biochemical, and multicellular environment of the joint. Moreover, the present findings were obtained using chondrocytes derived from non-diseased donors exposed to a single concentration of IL-1β, and therefore reflect the response to an IL-1β-induced inflammatory model rather than the full multifactorial pathophysiology of osteoarthritis, which also involves aging, mechanical stress, synovitis, immune-cell infiltration, and the combined action of multiple inflammatory mediators; these findings should not be generalized to the disease as a whole without further validation in disease-relevant settings. In addition, chondrocytes maintained in two-dimensional monolayer culture have been reported to undergo some degree of dedifferentiation [76], which represents an inherent limitation of this experimental model. Nevertheless, this approach enabled controlled, time-resolved analysis of chondrocyte responses to inflammatory signaling. Another important limitation is that the chondrocytes used for proteomic analysis were derived from only 2 donors. The complementary molecular and cellular assays performed here, including immunofluorescence, qRT-PCR, proliferation assays, and conditioned-medium profiling, were carried out using chondrocytes from one of the same donors included in the proteomic experiments; therefore these assays should therefore be regarded as orthogonal corroboration of selected findings within this experimental model rather than independent biological validation. Several pathway-level observations, including CDK4/Cyclin D1 suppression, mitochondrial and endoplasmic reticulum-associated remodeling, and changes in autophagy- and lysosome-related proteins, were not directly assessed by dedicated functional assays and will require confirmation in independent donor-derived cultures. Future studies involving larger and more diverse donor cohorts, ideally incorporating culture systems that better preserve the native chondrocyte phenotype and single-cell transcriptomic and proteomic approaches, will be required to resolve the heterogeneity of inflammatory and senescence-associated chondrocyte states in vivo.

## 5. Conclusions

Taken together, our findings support a model in which prolonged IL-1β exposure is associated with a coordinated transition from an acute inflammatory response toward a growth-arrested and extensively remodeled cellular state. This state is characterized by impaired proliferation, downregulation of components of the CDK4-Cyclin D1 signaling pathway, cytoskeletal reorganization, adaptation of organellar proteomes, and acquisition of transient senescence-associated traits. Importantly, the partial recovery of BrdU incorporation following cytokine withdrawal suggests that this phenotype could retain some degree of plasticity and may not fully correspond to a canonical irreversible senescence program, even though this observation does not establish reversibility of the broader phenotype or exclude a more persistent cellular state. Although the present study was conducted using chondrocytes derived from a limited number of donors, the consistency of the observed temporal patterns across multiple orthogonal experimental approaches, including DIA proteomics, immunofluorescence, qRT-PCR, proliferation assays, and targeted measurement of MMPs in conditioned medium, supports the internal consistency of the systems-level proteomic remodeling described here.

## Data Availability

All mass spectrometry proteomics data have been deposited to Mass Spectrometry Interactive Virtual Environment (MassIVE) Identifier: MSV000102314.

## Supplementary Materials

The following supporting information can be downloaded at https://www.mdpi.com/article/10.3390/cells15161431/s1, Table S1: Primer sequences used for qRT-PCR analyses. Supplementary Figure S1. Temporal abundance profiles of p16INK4a and LMNB1 during sustained IL-1β exposure. Data are presented as box-and-whisker plots with individual data points overlaid. Boxes represent the interquartile range (IQR), the center line indicates the median, and whiskers extend to the minimum and maximum values. Individual observations are displayed with horizontal jitter. Statistical significance is indicated by asterisks. Limma modeling with false-discovery rate correction was used. * *p*-value < 0.05. Supplementary Figure S2. Sustained IL-1β treatment induces down-regulation of COL2A1 in chondrocytes during the entire experimental course. Data are presented as box-and-whisker plots with individual data points overlaid. Boxes represent the interquartile range (IQR), the center line indicates the median, and whiskers extend to the minimum and maximum values. Individual observations are displayed with horizontal jitter. Statistical significance is indicated by asterisks. Limma modeling with false-discovery rate correction was used. * *p*-value < 0.05. Supplementary Figure S3. Sustained exposure to IL-1β (10 ng/mL) for 96 h induces increased secretion of pro-inflammatory mediators in chondrocytes. Data are presented as box-and-whisker plots with individual data points overlaid. Boxes represent the interquartile range (IQR), the center line indicates the median, and whiskers extend to the minimum and maximum values. Individual observations are displayed with horizontal jitter. The values in the y-axis represent concentrations in pg/mL. Controls were kept in culture for 96 h, while treated cells were exposed to IL-1β for 96 h with daily medium renewal and cytokine replenishment. Supplementary Spreadsheet S1: Differential Protein Expression Analysis across the Experimental Time Course—Quantitative proteomic data for all proteins identified by DIA-MS, including UniProt accession, gene symbol, protein name, log_2_ fold change, adjusted *p*-value (limma), and LFQ intensity values for each culture-level replicate. Pairwise comparisons were performed between IL-1β-treated samples (24 h, 48 h, 72 h, and 96 h) and untreated control cells maintained under contact inhibition for 96 h. Supplementary Spreadsheet S2: Gene Ontology Enrichment Analysis of Differentially Regulated Proteins—Complete Gene Ontology (Biological Process) enrichment results for differentially regulated proteins identified at each experimental time point. The spreadsheet includes enriched GO terms, associated genes, gene ratios, adjusted *p*-values, and enrichment statistics used to generate the functional analyses presented in Figure 1. Supplementary Spreadsheet S3: ANOVA Results for Proteins Differentially Regulated across the Experimental Time Course—Statistical results of the one-way ANOVA performed on proteins quantified across all experimental groups. The spreadsheet includes *p*-values, false discovery rate (FDR)-adjusted *p*-values, and associated protein annotations. Proteins with FDR < 0.05 were subsequently used for hierarchical clustering analyses presented in Figure 2. Supplementary Spreadsheet S4: Protein Membership of Temporal Expression Clusters—Complete lists of proteins assigned to each of the six temporal expression clusters identified by hierarchical clustering of significantly regulated proteins, as presented in Figure 2. Cluster assignment, protein identifiers, gene symbols, and protein names are provided.

## Abbreviations

The following abbreviations are used in this manuscript:

ADAMTS	A Disintegrin and Metalloproteinase with Thrombospondin Motifs
ANOVA	Analysis of Variance
ATP	Adenosine Triphosphate
BrdU	Bromodeoxyuridine
CDK4	Cyclin-Dependent Kinase 4
COMP	Cartilage Oligomeric Matrix Protein
COPI	Coat Protein Complex I
COPII	Coat Protein Complex II
DIA	Data-Independent Acquisition
ECM	Extracellular Matrix
ER	Endoplasmic Reticulum
FDR	False Discovery Rate
GO	Gene Ontology
HCS	High Content Screening
IL-1β	Interleukin-1β
LC-MS/MS	Liquid Chromatography–Tandem Mass Spectrometry
LFQ	Label free quantification
LMNB1	Lamin B1
MCM	Minichromosome Maintenance
MMP	Matrix Metalloproteinase
NHAC-kn	Normal Human Articular Chondrocytes, Knee-Derived
OXPHOS	Oxidative Phosphorylation
PCA	Principal Component Analysis
qRT-PCR	Quantitative Reverse Transcription Polymerase Chain Reaction
RHOA	Ras homolog family member A
ROCK2	Rho-associated protein kinase 2
SA-β-gal	Senescence-Associated β-Galactosidase
TNFα	Tumor Necrosis Factor Alpha
UPR	Unfolded Protein Response
V-ATPase	Vacuolar-Type ATPase
