# Supplementary material for: Proteomic Dynamics Reveal Cell-Cycle and Rho GTPase Remodeling Associated with Transient Senescence Traits in Human Chondrocytes During Sustained IL-1β Signaling
Source: Cells. 2026 Aug 8;15(16):1431. doi: 10.3390/cells15161431 (PMC13511605; doi:10.3390/cells15161431)
Supplement: Supplementary file 1 [file cells-15-01431-s001.zip › Supplementary Figures and Table.pdf]

Supplementary File Containing Supplementary Figures and Table

Proteomic Dynamics Reveal Cell-Cycle and Rho GTPase Remodeling Underlying Senescence-Associated Traits in Human Chondrocytes during Sustained IL-1beta Signaling

Hellen Paula Valerio *, Thatiana Corrêa de Melo, Mariana Barbosa de Souza Rizzo, Amanda Teixeira de Melo, Miryam Paola Alvarez-Flores and Ana Marisa Chudzinski-Tavassi *

Centre of Excellence in New Target Discovery (CENTD), Butantan Institute, São Paulo 05503-900, Brazil
* Corresponding authors

Table S1: Primer sequences used for qRT-PCR analyses.

**Gene**	**Forward**	**Reverse**
CDKN2A	GAAGGTCCCTCAGACATC	CCCTGTAGGACCTTCGGTGAC
TP53	TAACAGTTCCTGCATGGGCGGC	AGGACAGGCACAAACACGCACC
RHOA	ACGTGCCCATCATCCTGGTT	GGCTCCTGCTTCATCTTGGC
RAC1	CAGATTACGCCCCCTATCCTATCC	GGATACCACTTTGCACGGACAT
RPL37A	ATTGAAATCAGCCAGCACGC	AGGAACCACAGTGCCAGATCC
B2M	TGACTTTGTCACAGCCCAAGATA	AATGCGGCATCTTCAAACCT

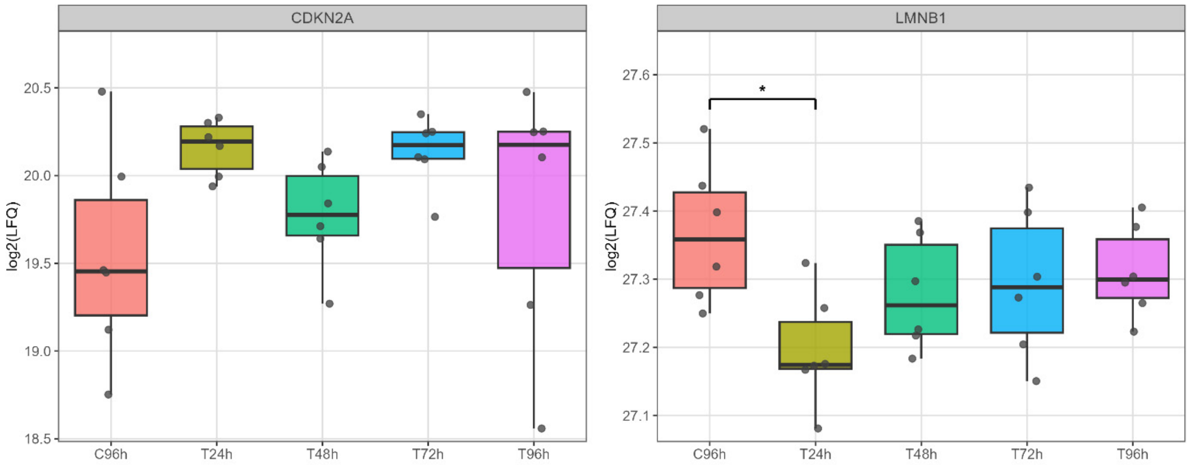

Supplementary Figure S1. Temporal abundance profiles of p16INK4a and LMNB1 during sustained IL-1β exposure. Data are presented as box-and-whisker plots with individual data points overlaid. Boxes represent the interquartile range (IQR), the center line indicates the median, and whiskers extend to the minimum and maximum values. Individual observations are displayed with horizontal jitter. Statistical significance is indicated by asterisks. Limma modeling with false-discovery rate correction was used. *p-value < 0.05.

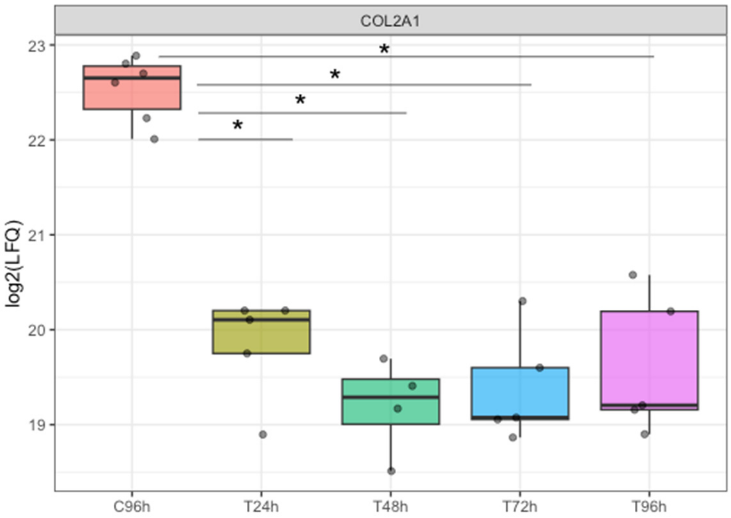

Supplementary Figure S2. Sustained IL-1β treatment induces down-regulation of COL2A1 in chondrocytes during the entire experimental course. Data are presented as box-and-whisker plots with individual data points overlaid. Boxes represent the interquartile range (IQR), the center line indicates the median, and whiskers extend to the minimum and maximum values. Individual observations are displayed with horizontal jitter. Statistical significance is indicated by asterisks. Limma modeling with false-discovery rate correction was used. *p-value < 0.05.

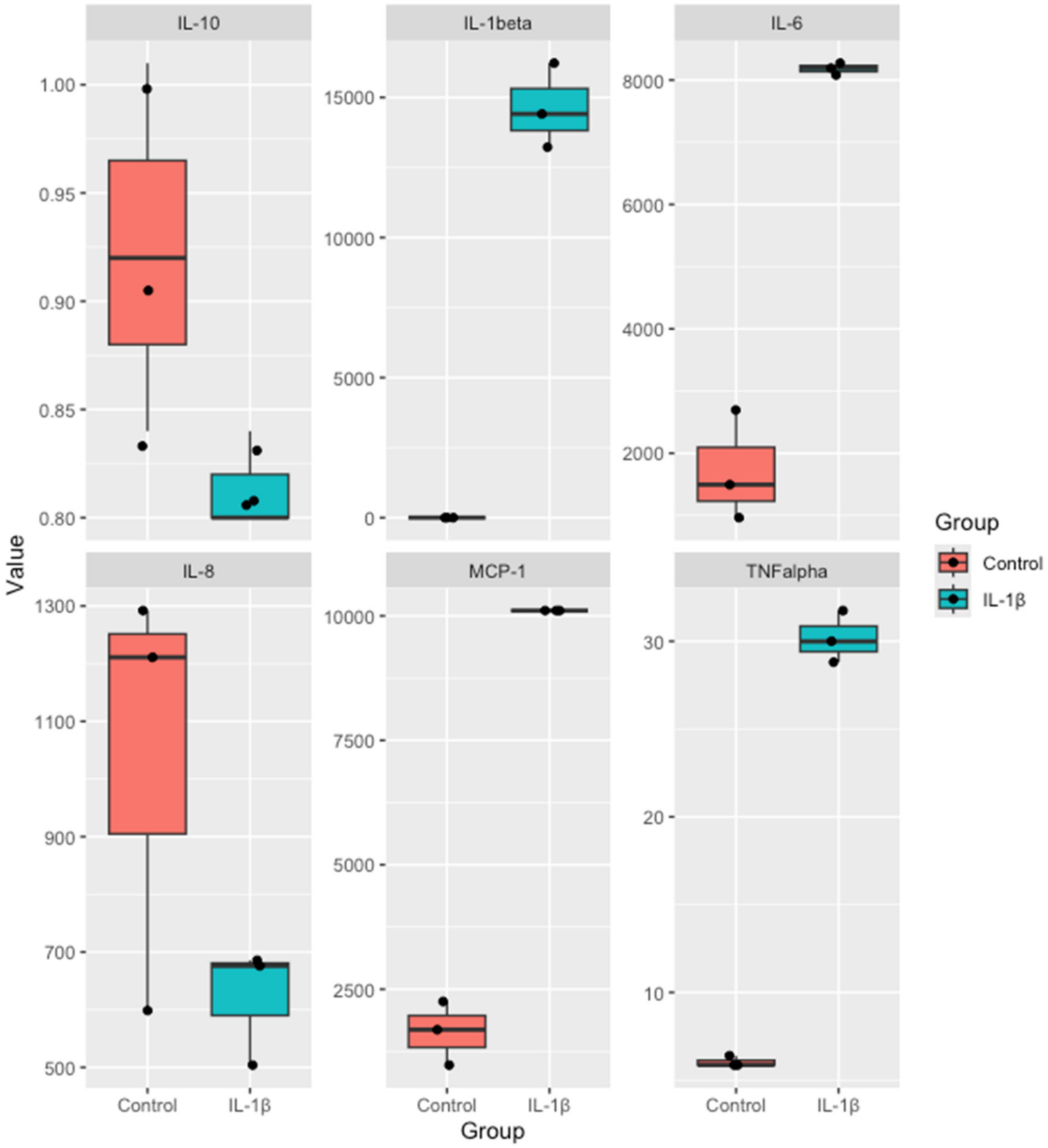

Supplementary Figure S3. Sustained exposure to IL-1β (10 ng/mL) for 96 hours induces increased secretion of pro-inflammatory mediators in chondrocytes. Data are presented as box-and-whisker plots with individual data points overlaid. Boxes represent the interquartile range (IQR), the center line indicates the median, and whiskers extend to the minimum and maximum values. Individual observations are displayed with horizontal jitter. The values in the y-axis represent concentrations in pg/mL. Controls were kept in culture for 96 hours, while treated cells were exposed to IL-1β for 96 hours with daily medium renewal and cytokine replenishment.
